# ZC3H15 in human tumors: a pan-cancer exploration of its clinical significance, molecular function, and association with the tumor immune microenvironment

**DOI:** 10.3389/fimmu.2026.1901516

**Published:** 2026-10-05

**Authors:** Bin Zhou, Jian Huang, Qifei Tao, Zhejin Sheng, Fabiao Zhang, Beige Jiang, Limei Li

**Affiliations:** 1 Department of Vascular Surgery, Shanghai East Hospital, Tongji University School of Medicine, Shanghai, China; 2 The Third Department of Hepatic Surgery, Eastern Hepatobiliary Surgery Hospital, Naval Medical University, Shanghai, China; 3 School of Life Science and Technology, Tongji University, Shanghai, China; 4 Key Laboratory of Minimally Invasive Techniques and Rapid Rehabilitation of Digestive System Tumor of Zhejiang Province, Department of Hepatobiliary Surgery, Taizhou Hospital of Zhejiang Province, Wenzhou Medical University, Linhai, Zhejiang, China; 5 Research Center for Translational Medicine, Shanghai East Hospital, School of Medicine, Tongji University, Shanghai, China

**Keywords:** cell cycle, diagnosis, pan-cancer analysis, prognosis, tumor immune microenvironment, zinc finger CCCH-type protein 15 (ZC3H15)

## Abstract

**Background:**

Zinc finger CCCH-type protein 15 (ZC3H15) is a ribosome-associated eukaryotic protein involved in many biological processes, including gene expression regulation, translation, and immune modulation. Although ZC3H15 has been implicated in the development of certain tumors, its expression, functional relevance, diagnostic potential, and prognostic value in a pan-cancer context remain not fully elucidated. This study conducts an integrative, systematic, and comparative analysis of ZC3H15 across 33 cancer types and subsequently provides further experimental validation in liver cancer.

**Methods:**

mRNA expression data of ZC3H15 were obtained from The Cancer Genome Atlas (TCGA) and Genotype-Tissue Expression (GTEx) datasets. Protein level validations were performed using the Human Protein Atlas (HPA) database and the Clinical Proteomic Tumor Analysis Consortium (CPTAC) database. The cBioPortal database was used to analyze the genomic alteration of ZC3H15. Two pathway enrichment analysis methods were conducted to explore the biological functions and molecular mechanisms of ZC3H15 in pan-cancer.

**Results:**

The results indicated that ZC3H15 was overexpressed in a wide range of tumors. In certain cancers, particularly LIHC, LUAD, BRCA, LUSC, and HNSC, its expression was significantly correlated with poor patient prognosis. Mutations and amplifications were the most common gene alterations in ZC3H15. The GSCA database showed that methylation levels were reduced in most tumor types. Abnormal expression of ZC3H15 was related to immune cell infiltration. Gene enrichment analysis indicated that ZC3H15-related genes involved in tumor progression were significantly enriched in multiple cell cycle processes. We demonstrated that knockdown of ZC3H15 suppressed cell proliferation, migration, and invasion in two liver cancer cell lines, MHCC97H and Huh7, and two BRCA cell lines, MDA-MB-231 and Hs 578T. To further clarify the functional mechanism, subsequent immunofluorescence staining confirmed that ZC3H15 knockdown induced prometaphase arrest in MHCC97H and Huh7 liver cancer cells.

**Conclusions:**

Overall, this pan-cancer study demonstrates that ZC3H15 serves as a potential biomarker for cancer diagnosis, prognosis, and immune cell infiltration.

## Introduction

Cancer is a complex and heterogeneous disease and remains a leading cause of global mortality (1–3). Cancers are traditionally classified by their tissue of origin (1). However, significant differences exist in incidence rates, mortality rates, and molecular mechanisms among different cancer types (1). Despite these differences, cancers share common characteristics, including key molecular pathways and therapeutic targets (1, 4). Therefore, reclassifying cancer subtypes based on molecular features is essential, as it enables more precise diagnosis and targeted therapies. Pan-cancer studies compare various cancer types, focusing on their shared characteristics to uncover universal mechanisms of carcinogenesis (5, 6). Pan-cancer analysis can identify diagnostic or prognostic markers that are universal across cancers, such as circulating tumor DNA and tumor mutation burden (TMB), which aid early cancer screening and help predict treatment response (7, 8).

Zinc finger CCCH type containing 15 (ZC3H15) is a highly conserved ribosome-associated eukaryotic protein, widely expressed in various human tissues and involved in multiple cellular biological processes, including gene expression regulation, translation, and signal transduction (9). ZC3H15 promotes tumor cell proliferation and metastasis. In hepatocellular carcinoma, ZC3H15 interacts with TNF receptor-associated factor 2 (TRAF2) to activate the nuclear factor-κB (NF-κB) signaling pathway (10). This interaction promotes tumor growth and vascular invasion, and correlates with elevated serum Alpha-fetoprotein (AFP) levels. Hepatocellular carcinoma patients with high expression of ZC3H15 are more prone to tumor metastasis and recurrence after curative treatment (10). In gastric cancer, high ZC3H15 expression increases c-Myc stability by inhibiting F-box and WD repeat domain containing seven (FBXW7) transcription, thereby promoting cell proliferation, migration, and invasion (11). In glioblastoma multiforme (GBM), ZC3H15 stabilizes the epidermal growth factor receptor (EGFR) protein by downregulating Casitas B-lineage (CBL) expression, reducing its ubiquitin-mediated degradation, thereby activating the EGFR signaling pathway and promoting tumor cell proliferation, migration, and *in vivo* tumor formation (12). In acute myeloid leukemia, ZC3H15 is significantly overexpressed and interacts with tumor necrosis factor receptor associated factor 2 (TRAF2) to affect the NF-κB pathway, which may serve as a key regulatory factor in tumorigenesis (13). Compared with normal tissues, ZC3H15 is highly expressed in melanoma tissues, and elevated ZC3H15 expression is associated with poorer survival in melanoma patients (14). Taken together, ZC3H15 promotes tumor initiation and progression, and is associated with poor prognosis in various tumors.

ZC3H15 not only regulates tumor cell growth, proliferation, and migration, but also participates in some immune responses. ZC3H15 is closely associated with oxidative stress and inflammatory processes. In the bone cancer pain (BCP) model, ZC3H15 regulates the Kelch-like ECH-associated protein 1 (KEAP1)/nuclear factor erythroid 2-related factor 2 (NRF2) pathway, which impairs neuronal antioxidant defense (15). Moreover, it inhibits the activation of microglial inflammation via the nuclear factor kappa B inhibitor alpha (IκBα)/NF-κB pathway. Additionally, ZC3H15 can influence NF-κB signaling and mitogen-activated protein kinase (MAPK) activity by interacting with TRAF2, an important adaptor protein involved in pro-inflammatory toll-like receptors (TLR) signaling in macrophages (10, 13). This interaction contributes to the regulation of inflammatory responses.

Previous studies have shown that the abnormal expression of ZC3H15 is involved in tumor cell growth, inflammatory response, and oxidative stress. However, comparative studies on its associations across distinct malignancies remain scarce, particularly regarding the diagnostic and prognostic potential from a pan-cancer perspective. We employed a pan-cancer approach to elucidate the commonalities, differences, and novel associations of ZC3H15 across a broad spectrum of cancers. We analyzed the expression of ZC3H15 in various types of cancer. Furthermore, we conducted univariate Cox regression and Kaplan-Meier survival analyses to evaluate the effects of ZC3H15 on overall survival (OS), disease-specific survival (DSS), and progression-free interval (PFI). We also investigated the correlations between ZC3H15 expression and several clinical features, including TMB, microsatellite instability (MSI), and the tumor microenvironment. We performed pathway enrichment analysis to explore the underlying mechanisms of ZC3H15 and subsequently validated these findings in two hepatoma cell lines, MHCC97H and Huh7. ZC3H15 expression correlated with several clinical features in multiple malignant tumors. In some cancers, higher expression predicted poorer OS, PFI, and DSS, and was also associated with infiltration of various immune cells. This study indicates that ZC3H15 may be a valuable biomarker for tumor diagnosis, immune cell infiltration, and prognosis.

## Materials and methods

### ZC3H15 mRNA data collection and differential expression analysis in 33 cancer types

The gene expression data used in this study were obtained from The Cancer Genome Atlas (TCGA) database (https://portal.gdc.cancer.gov/) and the Genotype-Tissue Expression (GTEx) database (https://commonfund.nih.gov/gtex). The TCGA database is sourced from more than 10,000 patients who represent 33 cancer types; their full names are shown in **Supplementary Table S1**. ZC3H15 expression data were transformed by applying the log2(transcripts per million (TPM)+1) transformation (16). Relevant clinical information, including OS, PFI, DSS, and clinical parameters, was also downloaded. When selecting samples from the TCGA and GTEx databases, we included all available tumor samples and corresponding normal tissue samples and excluded samples lacking complete clinical information or basic pathological data. Moreover, to ensure data consistency and comparability, we excluded samples that did not meet quality standards. Subsequently, differential expression analysis of ZC3H15 was performed between tumor and normal tissue samples in these cancer types leveraging integrated data from TCGA and GTEx. The Wilcoxon rank sum test was used to evaluate the statistical differences in expression between tumors and normal tissues in cancer datasets.

To validate ZC3H15 expression initially identified in the TCGA cohort, we downloaded ZC3H15 expression datasets for liver hepatocellular carcinoma (LIHC, GSE14520) (17, 18), lung adenocarcinoma (LUAD, GSE32863) (19), breast invasive carcinoma (BRCA, GSE15852) (20), lung squamous cell carcinoma (LUSC, GSE74706) (21), and head and neck squamous cell carcinoma (HNSC, GSE30784) (22) from the Gene Expression Omnibus (GEO) database, and conducted a differential expression analysis of ZC3H15 between the normal and tumor groups.

### Analysis of ZC3H15 protein expression

Data on ZC3H15 protein levels were obtained from human protein atlas (HPA) (23) (https://www.proteinatlas.org/) and UALCAN (24) (https://ualcan.path.uab.edu/) databases to examine its expression levels in normal human tissues and cancer tissues.

### The relationship between ZC3H15 expression and prognosis and diagnosis

This study applied the Cox proportional hazards model to evaluate the impact of individual factors on OS, DSS, and PFI in cancer prognosis. The survival package in R was used for univariate Cox survival analysis. For each type of cancer, the hazard ratio (HR) and its 95% confidence interval (CI) were calculated. The statistical significance of the difference in survival outcomes between the high expression group and the low expression group was evaluated by the log-rank test using the survfit function. Kaplan-Meier survival curves were generated to further analyze the correlation between ZC3H15 mRNA expression and cancer prognosis. The survival package in R was also used for survival analysis, and the optimal cutoff point was determined using the “surv_cutpoint” function in the survminer package. The survival package was employed to test the proportional hazards assumption and perform Cox regression analysis, while the rms package was utilized for model calibration and visualization. The diagnostic value of ZC3H15 in human cancer was studied through receiver operating characteristic (ROC) curve analysis (25). This analysis used the pROC package in R language to calculate the 95% confidence interval, area under the curve (AUC), and generated a smooth ROC curve.

### Multivariate Cox regression analysis

A multivariate Cox regression analysis was performed to evaluate prognosis. Specifically, the detailed procedure was described below: data preparation and cleaning involve organizing survival time (Time), outcome events (Status, where 1 indicates occurrence and 0 indicates censoring), and candidate variables. Prior to univariate analysis, samples with missing values in the outcome or time variables were excluded, as incomplete records cannot be included in the analysis. The variable screening strategy entailed conducting univariate Cox analysis on candidate variables. Those with P < 0.1 or established clinical significance were selected for the multivariate model, thus mitigating the risk of overfitting inherent in the simultaneous inclusion of all candidates. Multivariate models were constructed by incorporating screening variables into the `coxph(Surv(time, status) ~ var1 + var2 +…)` function; if the number of candidate variables exceeded 10, stepwise regression (forward, backward, or bidirectional) was used to optimize the model based on the Akaike Information Criterion (AIC) or Bayesian Information Criterion (BIC) and remove insignificant variables.

Key hypothesis testing: testing of the proportional hazards (PH) assumption via the `cox.zph` test must be performed, in which a P-value greater than 0.05 indicates that the assumption was satisfied; and multicollinearity must be assessed using Variance Inflation Factors (VIF), applying a threshold of 5 or 10, and exclude the affected variables if this threshold is exceeded. An HR > 1 indicates a risk factor, while an HR < 1 indicates a protective factor. Regarding the 95% confidence interval (CI), if the interval excludes 1, the result is considered statistically significant (P < 0.05). A P-value < 0.05 in the multivariate analysis identifies the variable as an independent prognostic factor, distinguishing it from a mere univariate association.

### Genetic map and expression pattern of ZC3H15 in pan-cancer

Using cBioPortal (https://www.cbioportal.org/) (26), we analyzed the mutation status of ZC3H15 in various types of cancer. We input “ZC3H15” into the “Quick Search” section of cBioPortal for cancer genomics. The “Cancer Type Summary” module was used to display the frequency of alterations across all tumor types represented in the TCGA database. Based on mutations in the genomic data, the TMB for each of the 33 tumor types was calculated using Perl scripts. To determine the number of mutated bases per million bases, the length of exonic regions used for TMB calculation was normalized. The MSI score was also obtained. Spearman’s rank correlation and the R function cor.test were employed to analyze the correlation between tumor gene expression, TMB, and MSI. Radar charts generated by the R package fmsb were used to visualize the TMB and MSI results. To evaluate the association between ZC3H15 methylation levels and cancer, we analyzed the correlation between methylation and gene expression levels using the Gene Set Cancer Analysis (GSCA) database (27).

### Correlation between ZC3H15 expression and the tumor immune microenvironment

Correlation between ZC3H15 expression and the tumor immune microenvironment was carried out as described previously (28). Based on ZC3H15 gene expression data, the ESTIMATE algorithm, implemented via the “estimate” and “limma” packages in R, calculated scores indicative of immune and stromal cell infiltration. These scores were used to estimate tumor purity and the levels of immune cells and extracellular matrix components in pan-cancer tissues. To further investigate immune cell infiltration across 33 types of malignant tumors, the single-sample Gene Set Enrichment Analysis (ssGSEA) algorithm was employed to assess the association between immune infiltration and ZC3H15 expression in TCGA tumors (29). Subsequently, a heatmap displaying the Pearson correlation coefficients between ZC3H15 expression and the abundance of various immune cell types was generated. This correlation was visualized using the “ggplot2” package (version 3.3.5) in R-Studio. Additionally, the association between ZC3H15 expression and immune cells in pan-cancer was analyzed using the TIMER2.0 database (https://cistrome.shinyapps.io/timer/) (30). To ensure result robustness, all correlation analyses between ZC3H15 expression and immune cell infiltration underwent correction for multiple comparisons using the Benjamini-Hochberg method to control the false discovery rate (FDR), resulting in adjusted P-values (adj. P).

### Gene set enrichment analysis

Patients from the TCGA cohort in each cancer type were stratified into high-expression and low-expression groups based on the expression levels of ZC3H15. Subsequently, the differentially expressed genes (DEGs) between these two groups were used for GSEA (31). Normalized Enrichment Score (NES) and FDR were calculated using the DEGs and the hallmark gene set with the R package GSEA (v1.38.2). The results were summarized and graphically presented in a bubble plot using the R package ggplot2 (v3.3.6). The R package “limma” was used for co-expression analysis, and the results were visualized using R packages “reshape2” and “RColorBrewer”.

### Gene Ontology and Kyoto Encyclopedia of Genes and Genomes pathway enrichment analysis

Based on the statistically significant association with ZC3H15 expression, LIHC, LUAD, BRCA, LUSC, and HNSC were selected for GO/KEGG pathway enrichment analysis. The pathway criterion for significant enrichment was an adjusted P-value < 0.05. The enriched pathways were visualized using the “ggplot2” software package (version 3.3.5) in R-Studio.

### Cell lines and cell culture

The human liver cancer cell line MHCC97H (RRID: CVCL_4972) was obtained from the Liver Cancer Institute, Fudan University, Shanghai, China. The Huh7 cell line (RRID: CVCL_0336) was purchased from the Cell Resources Center, Chinese Academy of Sciences, Shanghai, China. All cell lines were cultured in high-glucose DMEM supplemented with 10% fetal bovine serum (FBS) and penicillin-streptomycin (100 mg/L), and incubated at 37 °C in a humidified atmosphere with 5% CO_2_.

### RNA interference

siRNAs were purchased from Shanghai Saiheng Biotechnology (Shanghai, China). siRNAs were transfected using Lipofectamine™ RNAiMAX (Invitrogen, California, USA) according to the manufacturer’s protocols. MHCC97H and Huh7 liver cancer cells in the logarithmic growth phase were seeded one day prior to transfection. The original culture medium was replaced with fresh complete culture medium (without antibiotics) before transfection. Subsequently, 500 μL of the complex was slowly added to each well of a 6-well plate, gently mixed by agitation, and incubated in a 37 °C CO_2_ incubator. Eight hours later, the medium was replaced with complete culture medium to reduce toxicity.

The siRNAs used in this study were as follows:

siZC3H15#1: 5’ GAAGAUUAUCGAAGACAAAtt3’.
siZC3H15#2: 5’ GCAAAGCAACAGAAGUUUAtt3’.
siZC3H15#3: 5’ GGAUGACAAGAAGAAAGAAtt3’.
siNC: 5’ UUCUCCGAACGUGUCACGUTT3’.

### CCK-8 assay

MHCC97H and Huh7 cells were transfected with siRNAs for 12 hours and then seeded into 96-well plates containing 100 μL complete medium (1500 cells per well; six replicates), followed by incubation at 37 °C in an atmosphere of 5% CO_2_. At predetermined time points, CCK-8 reagent (Beyotime Biotechnology, Shanghai, China) was added to each well according to the manufacturer’s instructions. After a 3-hour incubation in the dark, absorbance values were measured at 450 nm using a microplate reader. The experiment was repeated three times.

### Transwell migration and invasion assays

For the migration assay, liver and breast cancer cells (2 × 10^5^ cells/200 μL) were seeded into Transwell chambers (8-μm pore size, Corning, Inc., Corning, NY, USA) and incubated for 24–48 hours. For the invasion assay, the upper chamber membranes of each Transwell insert were coated with a Matrigel basement membrane matrix (BD Biosciences), followed by the procedures outlined above for the migration assay. Cells that migrated through the Transwell membrane were fixed and stained with crystal violet, manually counted, and imaged.

### Cell cycle phase detection

Cell-cycle distribution was determined via flow cytometry analysis after the cells were stained with the PI/RNase Staining kit (Beyotime Biotechnology, Shanghai, China). According to the manufacturer’s instructions, cells were collected and washed twice with pre-cooled PBS. Pre-cooled 70% ethanol (-20 °C) was added, and the mixture was gently agitated. Cells were fixed overnight at -20 °C. The cells were centrifuged at 300 × g for 5 minutes and washed once with PBS. RNase A was added to achieve a final concentration of approximately 100 μg/mL. The mixture was incubated at 37 °C for 30 minutes to degrade RNA. Cells were stained with propidium iodide solution at room temperature in the dark for 30 min. DNA content was detected using flow cytometry.

### Western blot analysis

Cells were lysed using RIPA buffer (Beyotime Biotechnology, Shanghai, China) supplemented with protease inhibitors (Roche, Basel, Switzerland); the mixture was kept on ice and then centrifuged to collect the supernatant. The concentration was determined using the BCA method (Beyotime Biotechnology, Shanghai, China). Proteins were separated by 10% SDS-PAGE and subsequently transferred onto a 0.45 μm PVDF membrane at 388 mA. The membrane was blocked with 5% skim milk at room temperature for 1 hour. Then, the membrane was incubated with the diluted primary antibody against ZC3H15 (1:1000; ABclonal, Wuhan, China) overnight at 4 °C. β-actin (1:5000; Proteintech, Chicago, USA) was used as the loading control. After washing the membrane three times with TBST, the membrane was incubated with the HRP-conjugated secondary antibody (1:5000; Abcam, Cambridge, UK) at room temperature for 1 hour. ECL chemiluminescence substrate (Invitrogen, California, USA) was applied, and band signals were obtained by exposure using a chemiluminescence imaging system (Tanon 5200; Tanon, Shanghai, China).

### 5-ethynyl-2’-deoxyuridine incorporation assay

The EdU incorporation assay was employed to assess the efficiency of DNA replication. MHCC97H and Huh7 cell lines were seeded at a density of 1 × 10^5^ to 4 × 10^5^ cells per well in a 48-well plate and transfected with siRNA the following day. Three days post-transfection, the cells were incubated at 37 °C for 2 hours in complete medium containing 10 µM EdU (Bioagrio, Shanghai, China). Cells were fixed at room temperature with 4% paraformaldehyde (PFA) for 20 minutes. The cells were then incubated with the Click reaction mixture according to the manufacturer’s instructions. Cell nuclei were counterstained with DAPI. Images were captured using the THUNDER imaging system (Leica, Wetzlar, Germany).

### Immunofluorescence

Cells were fixed with 4% ice-cold PFA (Beyotime, Shanghai, China) for 20 minutes and permeabilized with 0.2% Triton X-100 (Sigma-Aldrich, Darmstadt, Germany) in PBS for 10 minutes, followed by a 30-minute blocking step in 1% BSA (Roche, Basel, Switzerland). The cells were then stained overnight at 4 °C with the following primary antibodies: Phospho-Histone H3 (Ser10) (1:1000, Starter Bio, Hangzhou, China), and α-tubulin (1:800, Proteintech, Chicago, USA). Next, the cells were incubated for 1 hour with the respective secondary antibodies conjugated to Alexa Fluor 488/555 (1:600; Huabio, Hangzhou, China), subsequently stained with DAPI (Beyotime, Shanghai, China) for an additional 10 minutes. Images were captured using a Leica Thunder fluorescent microscope and analyzed with open-source ImageJ software.

### Statistical analysis

The results were expressed as mean ± standard deviation (SD). Statistical analyses were conducted using R (version 4.4.0) or GraphPad Prism (version 9.0). Differences between two groups were assessed using either the Wilcoxon rank-sum test or Student’s t-test. Survival analyses were conducted using Kaplan–Meier curves, with differences assessed by the log-rank test. Correlations between variables were evaluated using Spearman’s rank correlation coefficient. Statistical significance levels were defined as follows: *P < 0.05, **P < 0.01, and ***P < 0.001.

## Results

### Expression of ZC3H15 gene in human cancers

Firstly, we analyzed ZC3H15 expression in normal tissues using the GTEx dataset. The results showed that ZC3H15 had low tissue specificity in normal tissues, indicating it was widely expressed across different tissues (**Figure 1A**). To evaluate the pan-cancer expression pattern of ZC3H15, we analyzed the ZC3H15 mRNA expression profiles of 33 different cancer types using the TCGA database. The expression level of ZC3H15 mRNA was elevated in various types of cancer, including BRCA, cholangiocarcinoma (CHOL), colon adenocarcinoma (COAD), esophageal carcinoma (ESCA), HNSC, LIHC, LUAD, LUSC, rectum adenocarcinoma (READ), and stomach adenocarcinoma (STAD) (**Figure 1B**). Notably, ZC3H15 was downregulated in cancers, such as kidney chromophobe (KICH), kidney renal papillary cell carcinoma (KIRP), kidney renal clear cell carcinoma (KIRC), and thyroid carcinoma (THCA).

**Figure 1:**
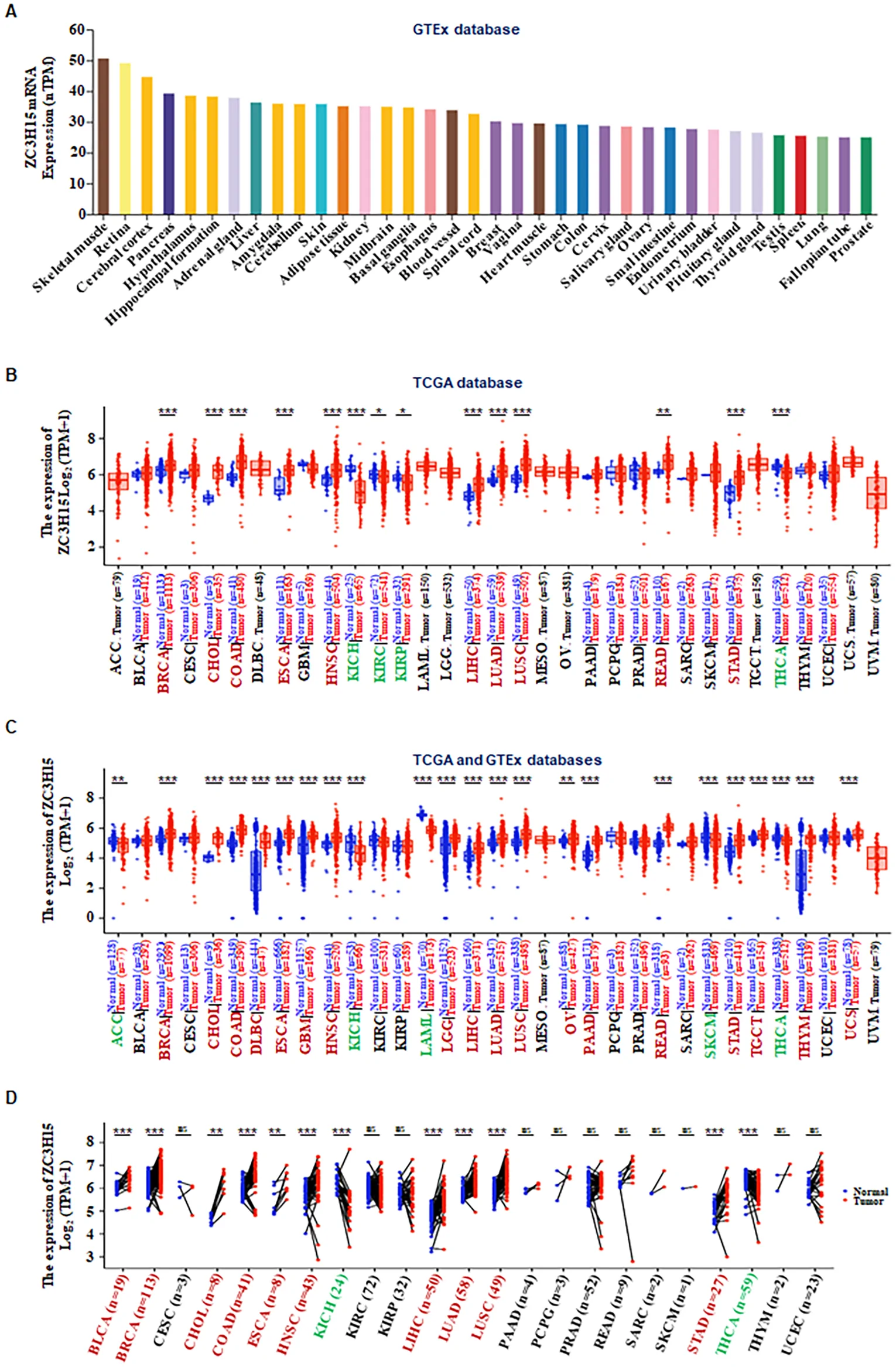
mRNA expression of ZC3H15 in 33 cancer types. **(A)** Expression levels of ZC3H15 mRNA in normal tissues from GTEx database. **(B)** Expression levels of ZC3H15 in normal and tumor tissues from the TCGA database. **(C)** Expression levels of ZC3H15 in normal and tumor tissues from the TCGA and GTEx databases. **(D)** Expression levels of ZC3H15 in tumors and adjacent normal tissues from the TCGA database. Statistical significance was indicated as follows: *P < 0.05, **P < 0.01, ***P < 0.001.

To further validate these findings, we combined the TCGA and GTEx datasets. ZC3H15 mRNA was elevated in multiple cancers, such as BRCA, CHOL, COAD, diffuse large B-cell lymphoma (DLBC), ESCA, HNSC, lower grade glioma (LGG), LIHC, LUAD, LUSC, ovarian serous cystadenocarcinoma (OV), pancreatic adenocarcinoma (PAAD), READ, and STAD (**Figure 1C**). Conversely, ZC3H15 expression was downregulated in adrenocortical carcinoma (ACC), KICH, acute myeloid leukemia (LAML), skin cutaneous melanoma (SKCM), and THCA (**Figure 1C**).

Pan-cancer paired expression analysis was also conducted, and the results showed that the expression of ZC3H15 was elevated in bladder urothelial carcinoma (BLCA), BRCA, CHOL, COAD, ESCA, HNSC, LIHC, LUAD, LUSC, READ, and STAD compared to normal tissues (**Figure 1D**). ZC3H15 was downregulated in KICH and THCA.

Collectively, these findings demonstrated that ZC3H15 was upregulated in various cancer types and downregulated in a few cancer types, such as KICH.

### Analysis of ZC3H15 expression and its correlation with clinical pathology in all cancer types

To investigate the relationship between ZC3H15 expression and the clinicopathological characteristics of various tumors, we evaluated ZC3H15 expression in patients at different pathological stages using UALCAN (**Figures 2A–O**). The expression of ZC3H15 showed a significant correlation with pathological stages in several cancers, including LIHC, LUAD, HNSC, and LUSC. ZC3H15 expression increased with higher pathological stages in LIHC, LUAD, HNSC, LUSC, uterine corpus endometrial carcinoma (UCEC), ACC, KIRP, and uveal melanoma (UVM) cancers. In mesothelioma (MESO), KICH, KIRC, OV, THCA, and testicular germ cell tumors (TGCT), it decreased with increasing pathological stages (**Figure 2**).

**Figure 2:**
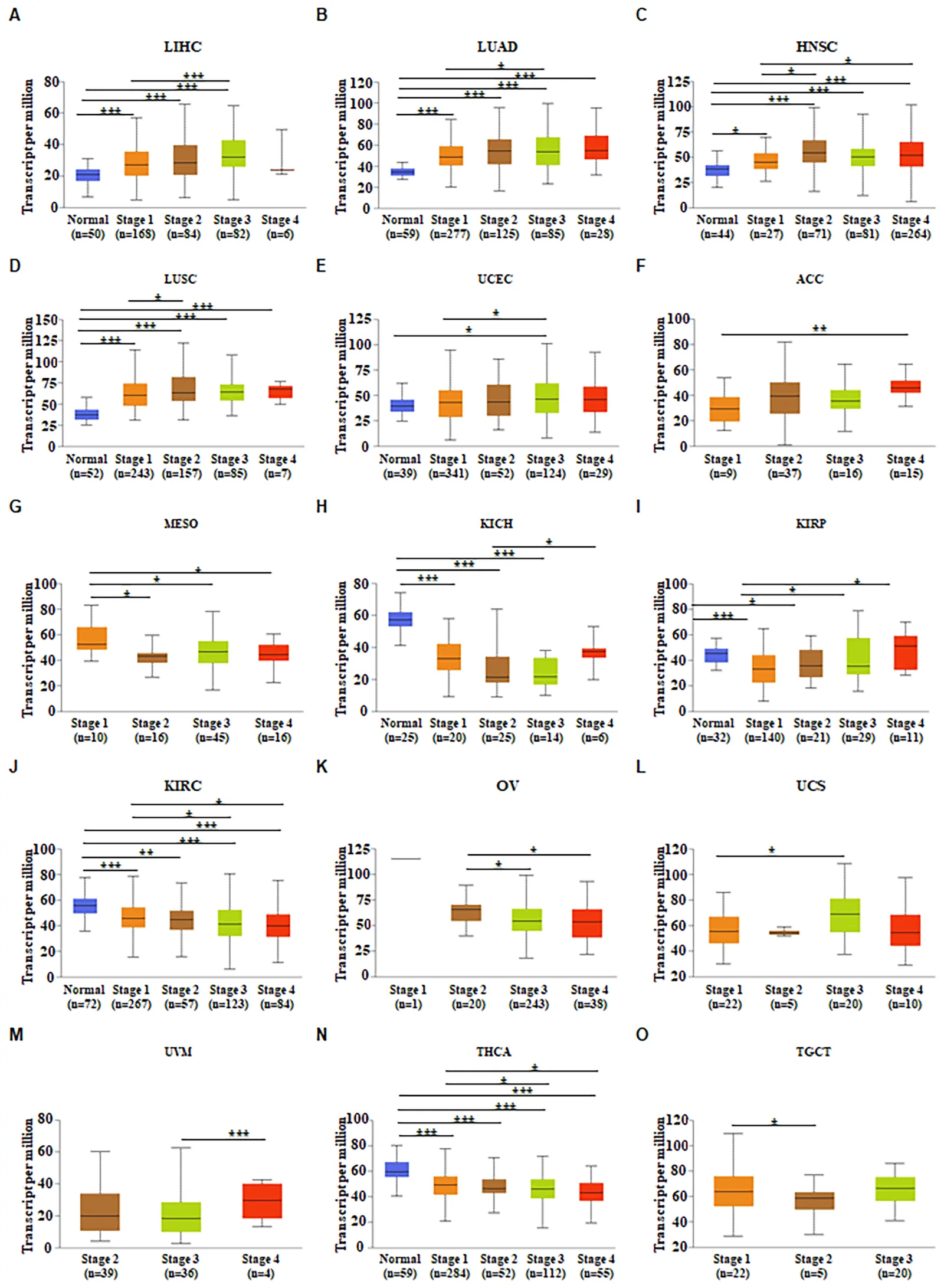
Correlation between ZC3H15 expression and clinical pathology in various cancer types. The relationship between ZC3H15 expression and clinical pathological stages I to IV in multiple cancer types, including LIHC **(A)**, LUAD **(B)**, HNSC **(C)**, LUSC **(D)**, UCEC **(E)**, ACC **(F)**, MESO **(G)**, KICH **(H)**, KIRP **(I)**, KIRC **(J)**, OV **(K)**, UCS **(L)**, UVM **(M)**, THCA **(N)**, TGCT **(O)**. Statistical significance was indicated as follows: *P < 0.05, **P < 0.01, ***P < 0.001.

The expression of ZC3H15 was significantly correlated with different tumor grades in various cancers. In LIHC, HNSC, PAAD, and STAD cancers, the expression of ZC3H15 increased with higher tumor grades. In KIRC, it decreased with increasing tumor grade (**Supplementary Figures S1A–F**).

The expression of ZC3H15 was also significantly correlated with nodal metastasis status in tumors. In LUAD, BRCA, LUSC, CHOL, and KIRP, it increased with higher nodal metastasis statuses (**Supplementary Figures S1G–K**). In conclusion, ZC3H15 demonstrated a positive correlation with pathological staging, specifically regarding lymph node metastasis, across certain tumor types.

### The diagnostic value of ZC3H15 and its impact on the prognosis of pan-cancer

To determine which tumors exhibit ZC3H15 expression levels capable of distinguishing between cancerous and normal tissues, ROC curve analysis was used to evaluate the diagnostic potential of ZC3H15 in pan-cancer, and the results showed that it has diagnostic potential for 13 types of cancer, including LIHC, LUAD, BRCA, HNSC, LUSC, READ, KICH, STAD, ESCA, COAD, CHOL, GBM, and THCA with accuracy (AUC>0.7) (**Figure 3A**). CHOL (AUC = 0.994) demonstrated particularly high accuracy. The AUC values for the remaining 12 cancers were: STAD (0.895), LUSC (0.89), ESCA (0.863), COAD (0.871), LIHC (0.83), LUAD (0.814), READ (0.778), BRCA (0.701), HNSC (0.715), GBM (0.716), KICH (0.715), and THCA (0.731) (**Figures 3B–N**). Notably, while the ROC curve can distinguish between tumors and normal tissues, it does not necessarily assess actual clinical diagnostic utility.

**Figure 3:**
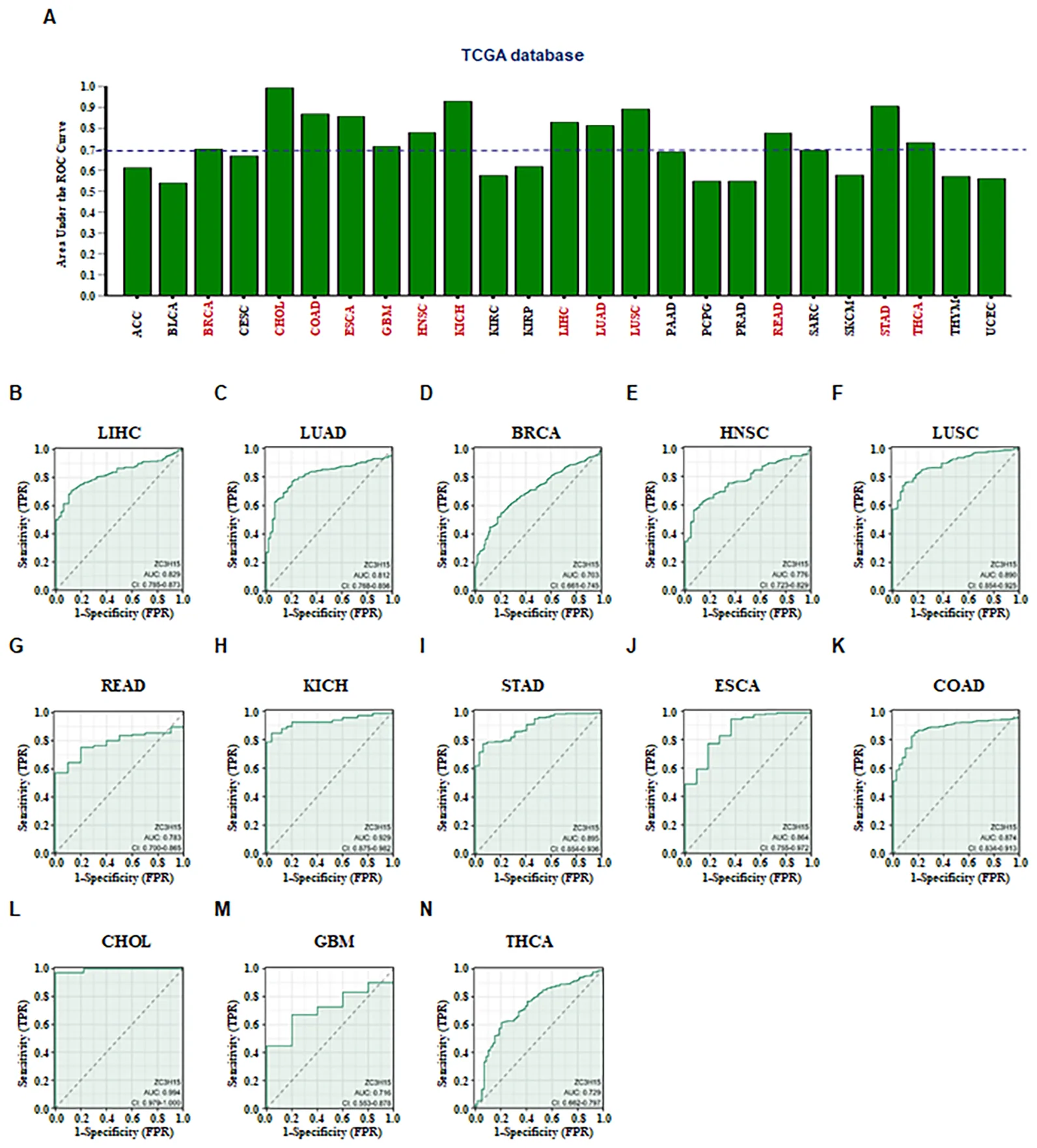
Diagnostic performance of ZC3H15 in cancer patients. **(A)** AUC of ROC curve used to evaluate the diagnostic performance of ZC3H15 in pan-cancer. ROC curves showing the diagnostic performance of ZC3H15 in multiple cancers: **(B)** LIHC, **(C)** LUAD, **(D)** BRCA, **(E)** HNSC, **(F)** LUSC, **(G)** READ, **(H)** KICH, **(I)** STAD, **(J)** ESCA, **(K)** COAD, **(L)** CHOL, **(M)** GBM, **(N)** THCA.

To examine the possible role of ZC3H15 in the survival and prognosis of tumor patients, we conducted univariate Cox regression and constructed a prognostic forest plot to evaluate the effects of ZC3H15 on OS, PFI, and DSS. The prognostic forest plot showed that ZC3H15 expression was significantly correlated with OS in 16 types of tumors: LIHC, LUAD, BRCA, PAAD, LGG, UCEC, prostate adenocarcinoma (PRAD), MESO, KIRP, READ, OV, uterine carcinosarcoma (UCS), cervical squamous cell carcinoma and endocervical adenocarcinoma (CESC), KICH, and ACC. High expression of ZC3H15 was associated with poor OS in LIHC, LUAD, BRCA, PAAD, LGG, CESC, UCEC, PRAD, MESO, KIRP, KICH, and ACC cancers and was a significant risk factor for these 12 types of cancer. In contrast, high expression of ZC3H15 in READ, OV, UCS, and KIRC, was associated with improved OS.

High ZC3H15 expression was significantly correlated with shorter PFI in LIHC, LUAD, HNSC, LUSC, PAAD, CESC, UCEC, PRAD, KIRP, KICH, and ACC (**Figure 4B**). However, high ZC3H15 expression was associated with improved PFI in OV, UCS, and KIRC (**Figure 4B**). In addition, high expression of ZC3H15 was significantly correlated with poor DSS in LIHC, LUAD, HNSC, PAAD, LGG, BLCA, CESC, UCEC, MESO, KIRP, KICH, and ACC, whereas DSS improved in OV and KIRC (**Figure 4C**). Kaplan-Meier survival analysis further confirmed these results, which showed tumor types that were significantly correlated with prognosis (**Figures 4D–F**).

**Figure 4:**
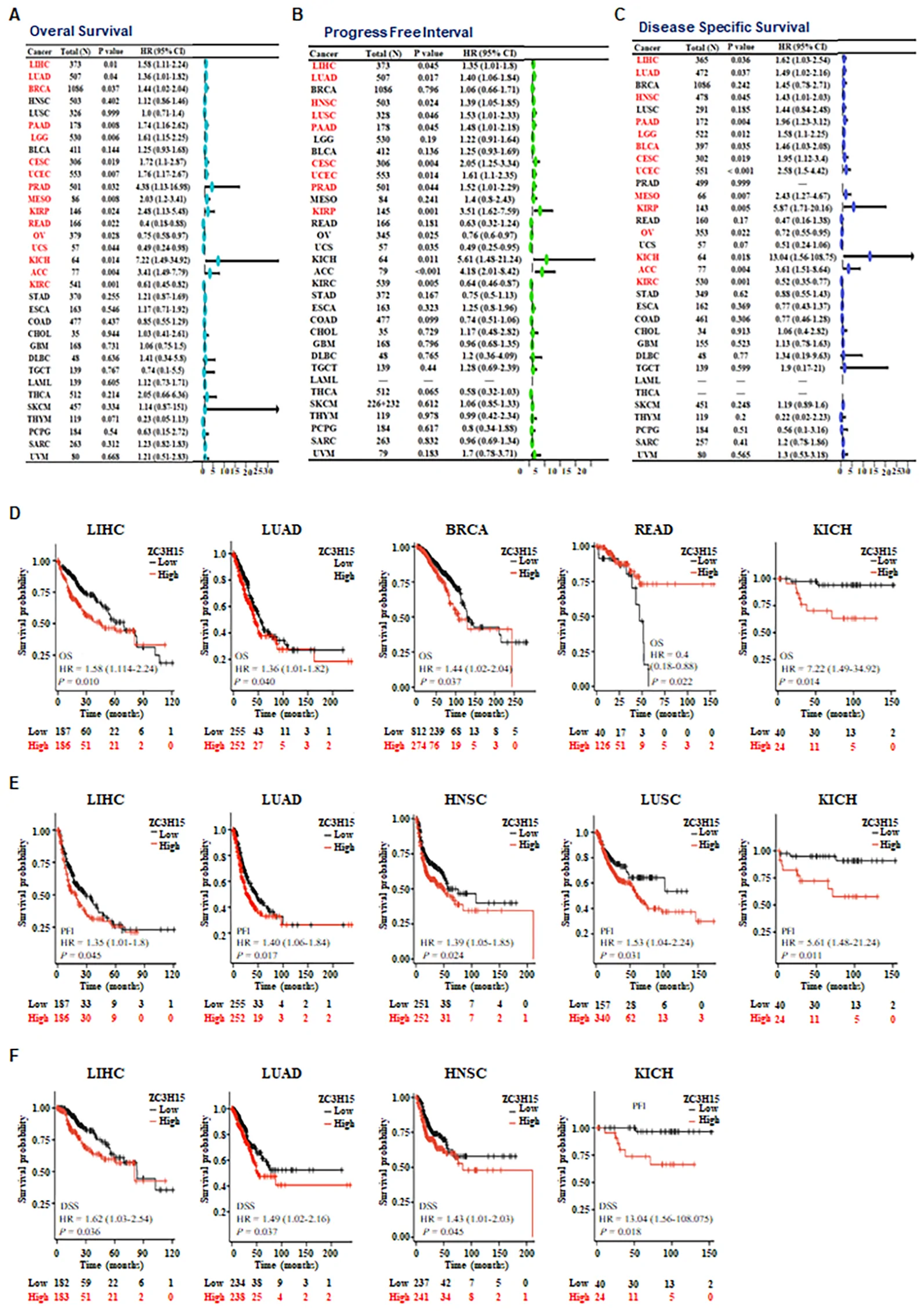
Correlation between ZC3H15 gene expression and cancer survival prognosis in various cancer types. Forest plot of different cancers. The red square represents tumor types with statistically significant correlation with prognosis. **(A)** Forest plot of the relationship between ZC3H15 expression and overall survival (OS). **(B)** Forest plot of the relationship between ZC3H15 expression and progression-free interval (PFI). **(C)** Forest plot of the relationship between ZC3H15 expression and disease-specific survival (DSS). **(D)** Kaplan-Meier survival curves showing differential expression of ZC3H15 in LIHC, LUAD, BRCA, READ, and KICH are presented. **(E)** Kaplan-Meier survival curves showing PFI in patients with differential expression of ZC3H15 in LIHC, LUAD, HNSC, LUSC, and KICH, respectively. **(F)** Kaplan-Meier survival curves of DSS in patients with differential expression of ZC3H15 in LIHC, LUAD, HNSC, and KICH, respectively. The red and blue lines represent high and low ZC3H15 expression groups, respectively. All P-values are shown in the figure. Only tumor types showing significant results and significant correlation with prognosis are presented.

The aforementioned prognostic analysis was limited to univariate Cox regression; however, tumor prognosis is generally confounded by clinical covariates such as age, gender, tumor stage and grade. To enhance the robustness of the findings, we used multivariate Cox regression across the LIHC, LUAD, BRCA, LUSC, and HNSC cohorts to evaluate whether ZC3H15 serves as an independent prognostic biomarker after adjusting for clinical covariates. Univariate Cox regression analysis demonstrated that pathological stage and tumor status were significantly associated with OS in LIHC, whereas age, sex, and BMI were not significantly associated with OS. Nevertheless, in light of the established clinical prognostic value of age, sex, and BMI in LIHC, these variables, together with pathological stage and tumor status, were included in the multivariate Cox regression model. The analysis demonstrated that ZC3H15 was an independent prognostic factor for OS in LIHC (HR = 1.747, 95% CI: 1.130–2.700; p = 0.012), as shown in **Supplementary Table 2**. Using the same analysis method, the results demonstrated that ZC3H15 was an independent prognostic factor for DSS in LIHC (HR = 1.662, 95% CI: 1.000 - 2.762; p = 0.050), as shown in **Supplementary Table 3**. However, ZC3H15 was not an independent prognostic factor for PFI in LIHC (HR = 1.089, 95% CI: 0.780–1.520; p = 0.617) (**Supplementary Table 4**).

ZC3H15 did not emerge as an independent prognostic factor for OS, PFI, or DSS within the LUAD cohort (**Supplementary Tables S5**–**7**). ZC3H15 demonstrated independent prognostic value for OS (HR = 1.679; 95% CI: 1.168–2.413; p = 0.005; **Supplementary Table 8**), but not for PFI or DSS in the BRCA cohort (data not showed). ZC3H15 in the LUSC cohort also demonstrated independent prognostic value for PFI (HR = 1.502; 95% CI: 1.018–2.214; p = 0.040; **Supplementary Table 9**), but not for OS or DSS (data not shown). In the HNSC cohort, while ZC3H15 was identified as an independent prognostic factor for both PFI (HR = 1.450; 95% CI: 1.079–1.950; p = 0.014; **Supplementary Table 10**) and DSS (HR = 1.462; 95% CI: 1.020–2.096; p = 0.039; **Supplementary Table 11**), it was not an independent predictor of OS.

Given the identification of ZC3H15 as an independent prognostic factor for specific endpoints across these cohorts (OS and DSS in LIHC; OS in BRCA; PFI in LUSC; and PFI and DSS in HNSC), six distinct nomograms incorporating ZC3H15 expression and clinical features specific to each cancer type were developed (**Supplementary Figures S2A–F**). To mitigate the risk of overfitting in the survival models, we assessed model robustness using calibration plots. Calibration plots were employed to evaluate the agreement between predicted 1-, 3-, and 5-year survival rates and observed outcomes, where the gray diagonal represents the line of perfect calibration. The closer the line of predicted values is to the diagonal, the better the goodness of fit. The calibration curves for 1-year, 3-year, and 5-year time points showed good agreement between the predicted and observed survival probabilities of the six nomograms (**Supplementary Figures S2G–L**).

In summary, ZC3H15 expression levels can effectively distinguish normal tissues from tumors across various tumor types. Furthermore, ZC3H15 expression was significantly associated with prognosis in certain tumor types.

### Protein levels and diagnostic value of ZC3H15 in human cancers

Proteins are key molecules linked to diseases, and changes in their levels directly relate to many pathological conditions. Further research on the differential expression of ZC3H15 protein between tumors and normal tissues is warranted. We examined the protein expression of ZC3H15 using the HPA dataset. ZC3H15 was widely expressed across various normal tissues and had a high expression level (**Figure 5A**). We analyzed the immunohistochemistry data in the HPA database to determine the presence of ZC3H15 protein in various cancer tissues. Strong positive staining was observed in some cancer tissues, while weak staining or no signal was detected in corresponding normal tissues (**Figure 5B**).

**Figure 5:**
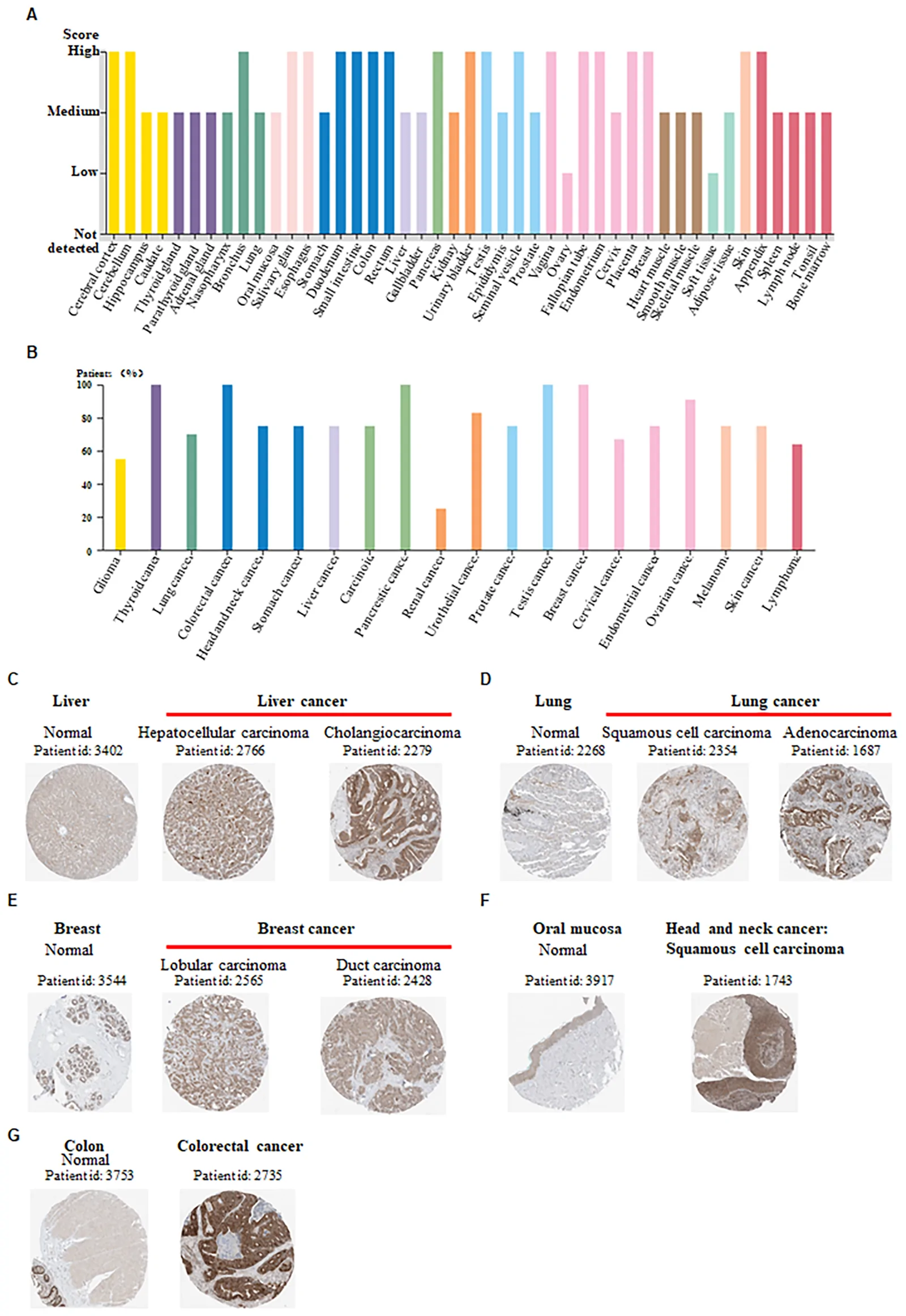
ZC3H15 protein expression in pan-cancer. **(A)** Expression levels of ZC3H15 protein in normal human tissues. **(B)** Expression levels of ZC3H15 protein in tumor tissues. Representative images showing ZC3H15 protein expression in normal and tumor tissues of liver cancer **(C)**, lung cancer **(D)**, breast cancer **(E)**, head and neck cancer **(F)**, and colorectal cancer **(G)**.

ZC3H15 mRNA expression was not only closely linked to the prognosis of LIHC, LUAD, BRCA, HNSC, and LUSC patients, but also provided relatively accurate diagnostic value for these patients (**Figures 3**, **4**). Therefore, we focused on presenting the expression of ZC3H15 in these five types of cancer: LIHC, LUAD, LUSC, BRCA, and HNSC. The results showed that compared with the corresponding normal tissues, ZC3H15 was upregulated in these five cancer types (**Figures 5C–G**).

Using the CPTAC database, we subsequently analyzed the protein expression of ZC3H15 in various tumor types. Our research results indicated that the protein level of ZC3H15 was significantly elevated in LIHC, LUAD, BRCA, HNSC, LUSC, COAD, GBM, UCEC, and OV, while significantly decreased in KIRC and pancreatic ductal adenocarcinoma (PDAC) (**Figure 6**; **Supplementary Figure S3**). Compared with normal tissues, ZC3H15 protein was upregulated in LIHC, LUAD, BRCA, HNSC, and LUSC, with elevated protein expression showing notable diagnostic value, particularly for BRCA (**Figure 6**).

**Figure 6:**
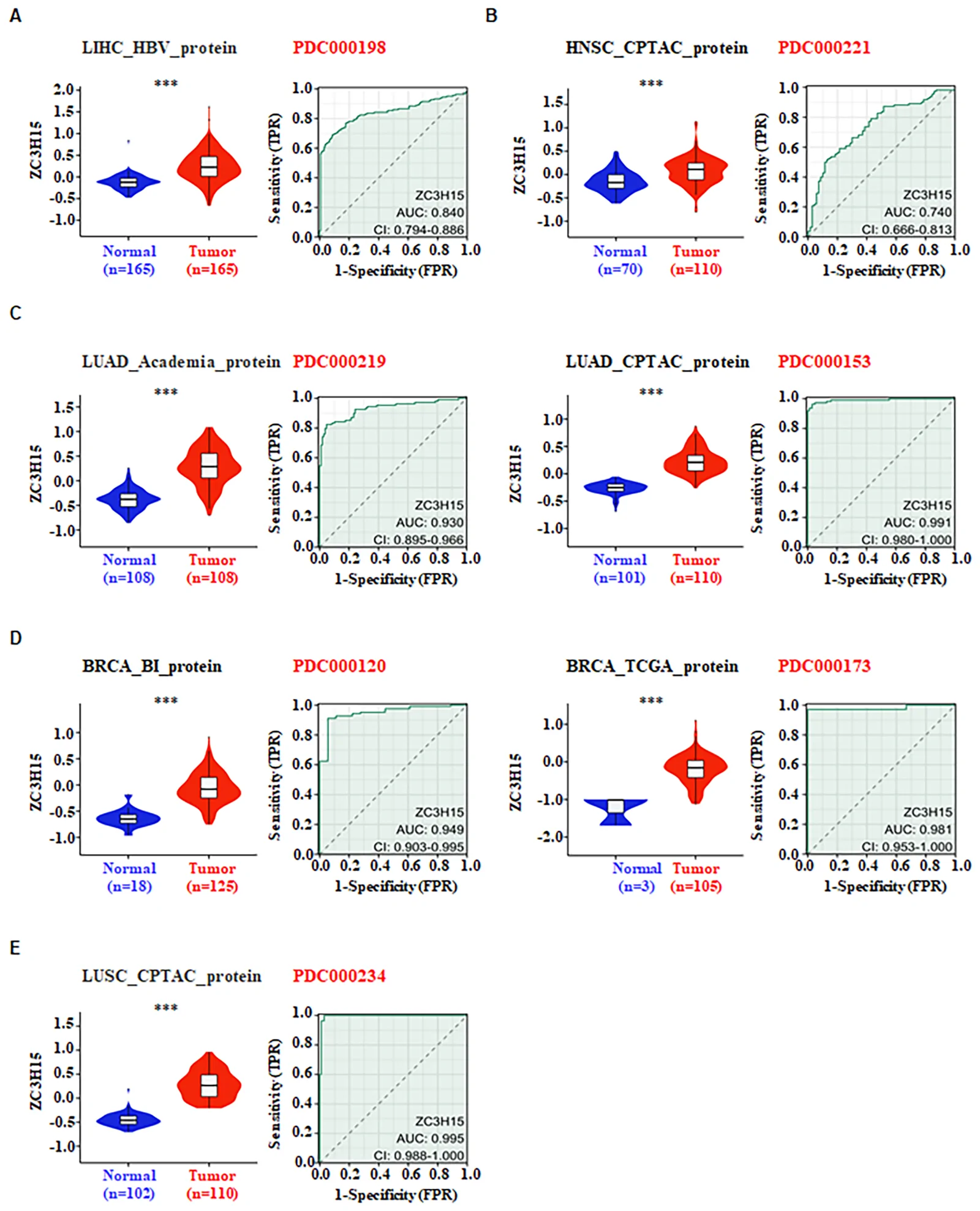
Detection of ZC3H15 protein expression levels and evaluation of its diagnostic value in cancer patients using the CPTAC dataset. **(A)** LIHC, **(B)** HNSC, **(C)** LUAD, **(D)** BRCA, **(E)** LUSC. *P < 0.05, **P < 0.01, ***P < 0.001.

### Genetic variation, copy number variations and methylation analysis of ZC3H15 in pan-cancer

Using the cBioPortal database, we analyzed the mutation frequency of ZC3H15 across 32 cancer types, as shown in **Figure 7A**. The results showed that ZC3H15 alterations in these 32 cancer types were mainly mutations and amplifications. ZC3H15 showed the highest mutation frequency of 5.48% in UCEC (**Figure 7A**). Further analysis revealed that ZC3H15 expression increased with copy number variations (CNVs), ranging from homozygous deletion to high-level amplification (**Figure 7B**). Analysis of TMB revealed that ZC3H15 exhibited a low mutation frequency in most cancers (**Figure 7C**). The relationship between ZC3H15 expression and TMB in various tumor types indicated that ZC3H15 expression was positively correlated with TMB in STAD, LUAD, and SKCM, but negatively correlated in THCA (**Figure 7C**). ZC3H15 expression was positively correlated with MSI in LUSC and STAD, while it was negatively correlated in DLBC and pheochromocytoma and paraganglioma (PCPG) (**Figure 7D**).

**Figure 7:**
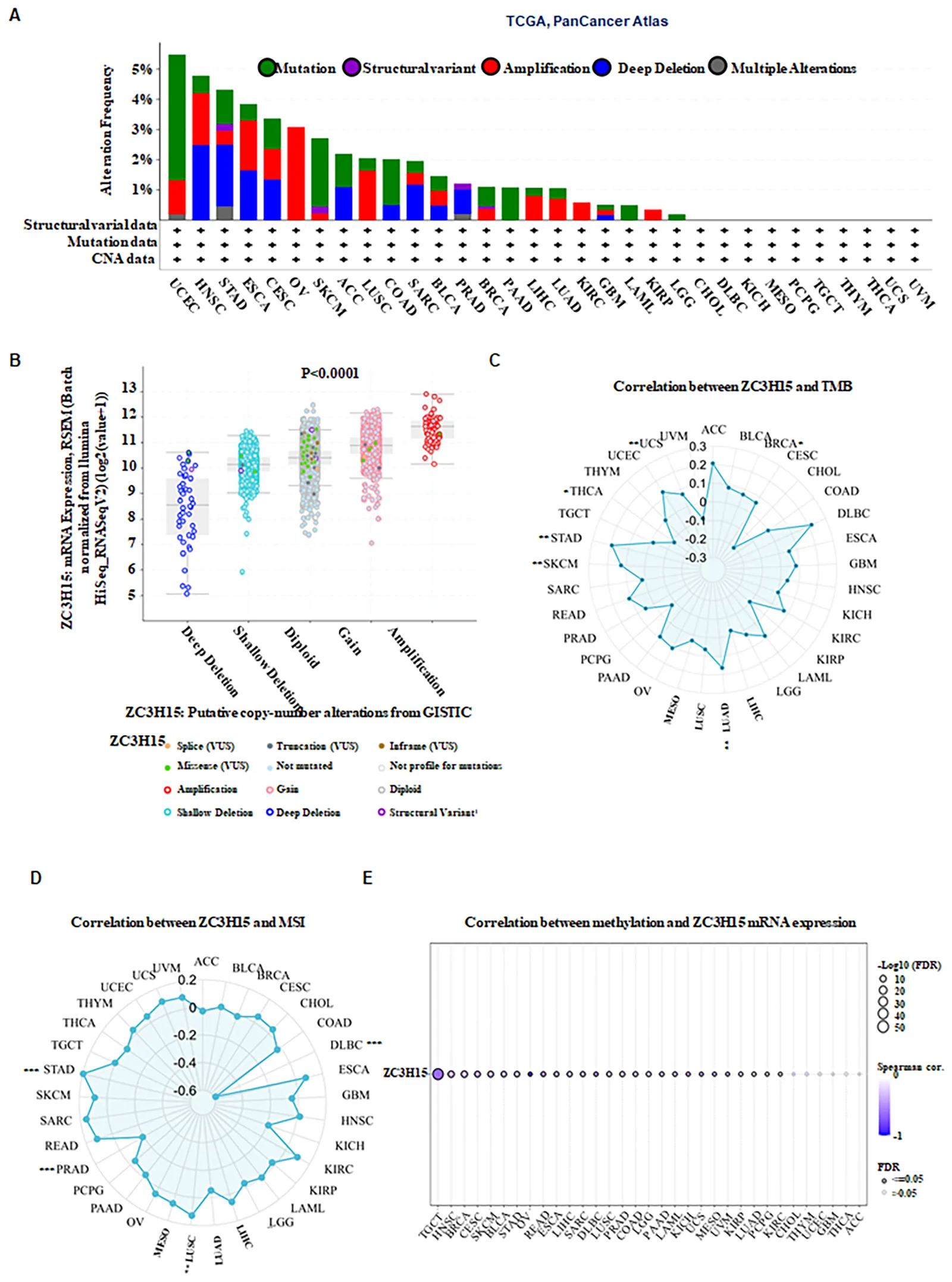
Analysis of genomic alterations and genomic instability in ZC3H15. **(A)** A pan-cancer analysis was performed on the genomic alterations of ZC3H15 in the TCGA database. The X-axis is grouped according to 33 types of cancer. The Y-axis represents the mutation frequency of ZC3H15 within each group. Colors represent the types of genomic alterations within each group. **(B)** This box plot combined with a point plot shows the correlation between differential expression of the ZC3H15 gene and ZC3H15 copy number variations across in 33 types of cancer. Radar charts represent the association analysis between ZC3H15 expression and TMB **(C)** and MSI **(D)**. *P < 0.05, **P < 0.01, ***P < 0.001. **(E)** The mRNA expression of ZC3H15 showed significant correlation with methylation levels.

The GSCA database was used to analyze the correlation between methylation and gene expression levels (**Figure 7E**). In most tumor types except CHOL, thymoma (THYM), UCEC, GBM, THCA, and ACC, ZC3H15 mRNA was significantly correlated with methylation levels. Among these, there was a strong negative correlation between methylation and expression in OV and TGCT. Weak negative correlations were observed in DLBC, READ, KICH, CESC, UCS, and ESCA (**Figure 7E**). Data from the UALCAN database indicated that ZC3H15 promoter regions exhibited hypomethylation in the vast majority of tumors (**Supplementary Figure S4**).

These findings suggest that ZC3H15 may play an important role in the pathogenesis of various cancers, influenced by copy number variations, promoter methylation, and genetic alterations. However, the TMB data indicated that there was no close association between ZC3H15 and overall genomic instability.

### The relationship between ZC3H15 gene and immune infiltration

We used the ESTIMATE method to investigate the association between ZC3H15 gene expression and tumor microenvironment across various cancer types. In most cancers, ZC3H15 expression showed a negative correlation with immune components of the tumor microenvironment (**Figure 8A**). Moreover, ZC3H15 expression was significantly negatively correlated with stromal score in pan-cancer, including TGCT, LUSC, and sarcoma (SARC) (**Figure 8A**).

**Figure 8:**
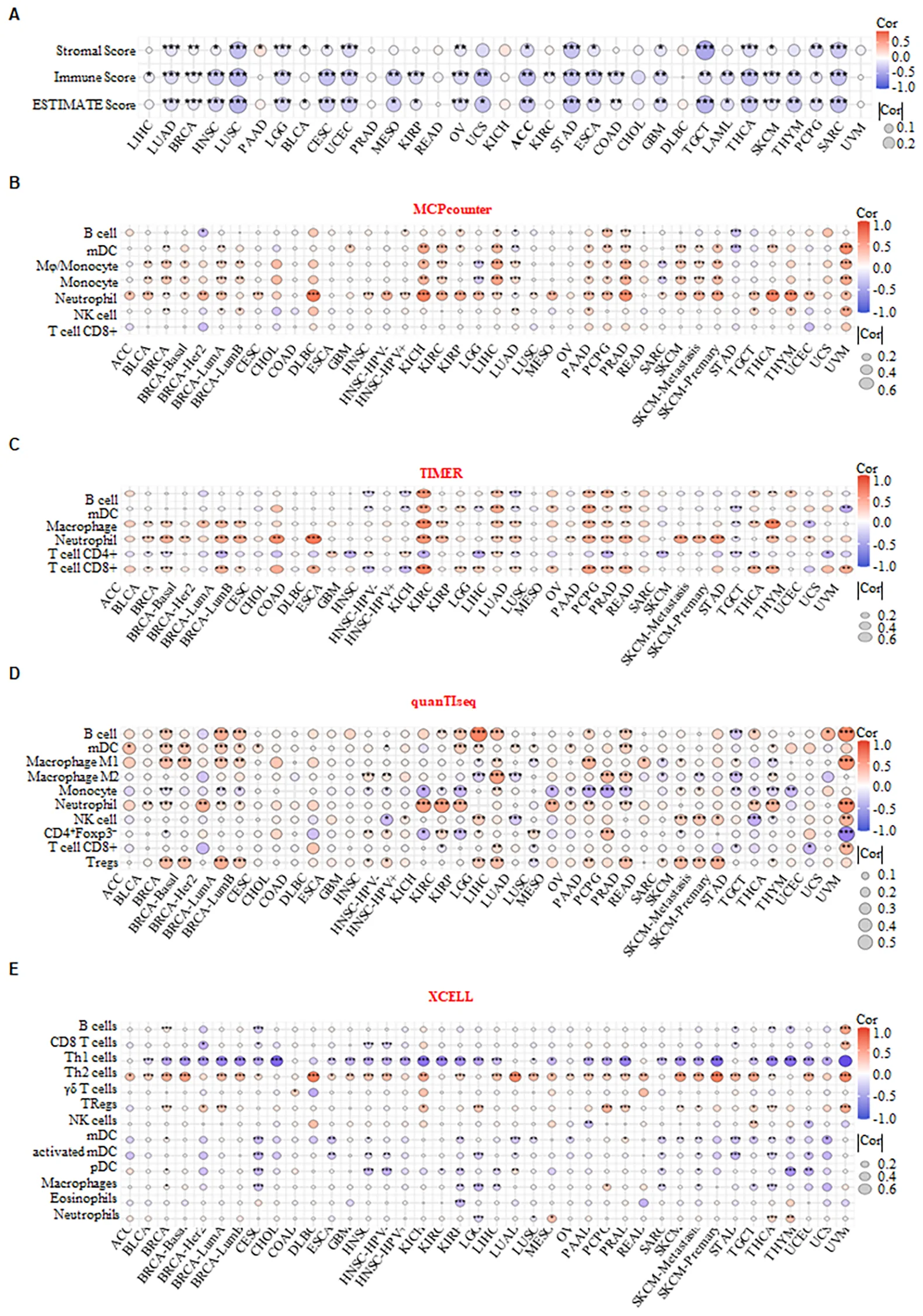
Correlation of ZC3H15 expression with immune cell infiltration in the tumor microenvironment. **(A)** The ESTIMATE algorithm evaluated the correlation between ZC3H15 expression and the stromal score and immune score. **(B)** The MCPcounter algorithm assessed the correlation between ZC3H15 and the infiltration of seven immune cell types in 33 cancer types. **(C)** The TIMER algorithm assessed the correlation between ZC3H15 expression and the infiltration levels of six immune cell types in 33 cancer types. **(D)** The quanTIseq algorithm assessed the correlation between ZC3H15 expression and the infiltration levels of 10 immune cell types in 33 cancer types. **(E)** The XCELL algorithm assessed the correlation between ZC3H15 expression and the infiltration of 13 immune cell types across 33 cancer types. *adj. P < 0.05, **adj. P < 0.01, ***adj. P < 0.001.

We subsequently employed multiple algorithms to evaluate the correlation between ZC3H15 expression and tumor immune cell infiltration (**Figures 8B–E**; **Supplementary Figures S5A–C**). The results revealed distinct correlations between ZC3H15 expression and immune cell infiltration across various tumor types. For instance, the application of the MCPcounter algorithm revealed that ZC3H15 expression was significantly correlated with numerous immune cell populations across cancers, exhibiting both positive and negative associations (**Figure 8B**). Specifically, in BRCA, ZC3H15 expression was positively correlated with neutrophils, macrophages, monocytes, myeloid dendritic cells, and natural killer (NK) cells. In HNSC, ZC3H15 expression was primarily positively correlated with neutrophils. In LIHC, ZC3H15 expression was positively correlated with neutrophils, macrophages, monocytes, myeloid dendritic cells, and B cells. In LUAD, ZC3H15 expression was primarily positively correlated with macrophages and monocytes. In LUSC, it was primarily positively correlated with neutrophils.

Notably, the MCPcounter, TIMER, and quanTIseq algorithms indicated that ZC3H15 was positively correlated with neutrophil infiltration across various tumors (**Figures 8B–D**). The MCPcounter and TIMER algorithms found a positive correlation between ZC3H15 and macrophage infiltration in various cancers (**Figures 8B, C**). Furthermore, analyses using the XCell and ssGSEA algorithms revealed that in most cancer types, ZC3H15 expression was positively correlated with Th2 cell infiltration and negatively correlated with Th1 cell infiltration (**Figure 8E**; **Supplementary Figure S5A**).

To investigate whether ZC3H15 recruits distinct immune cell subsets into tumors by modulating chemokine signaling pathways, we examined the correlations between ZC3H15 expression and that of chemokines and their receptors using the TIMER2.0 database. The results revealed a positive correlation between ZC3H15 and CXCL8 across 26 cancer types, including ACC, BRCA, HNSC, LIHC, LUAD, and THCA (**Figure 9A**). A positive correlation between ZC3H15 and CXCL10 was observed across 21 cancer types, notably in BRCA and LUAD (**Figure 9A**). ZC3H15 showed a positive correlation with CXCL11 across 21 cancer types, including BRCA, LIHC, and LUAD (**Figure 9A**). ZC3H15 was positively correlated with CCR1 and CCR8 in 25 types of cancer, including BRCA, LIHC, and LUAD (**Figure 9B**).

**Figure 9:**
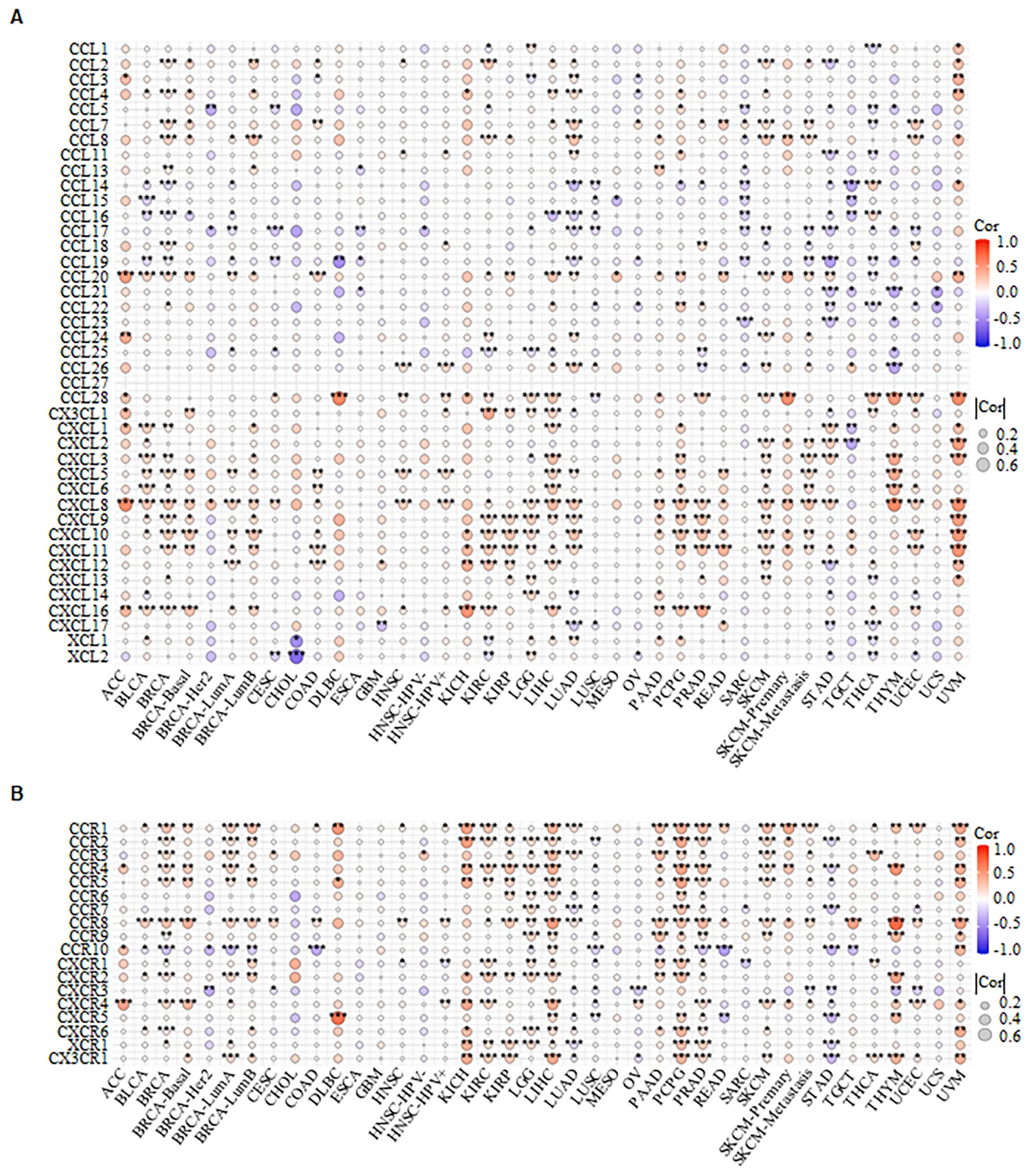
Correlation of ZC3H15 expression with chemokines **(A)** and their receptors **(B)** in pan-cancer. *adj. P < 0.05, **adj. P < 0.01, ***adj. P < 0.001.

The tumor immune microenvironment is a complex ecosystem composed of tumor cells, diverse immune cells, stromal cells, cytokines, and other signaling molecules (32). The dynamic balance between activating and suppressive immune factors within the tumor microenvironment is the primary determinant of the strength and persistence of anti-tumor immune responses. Disruption of this equilibrium directly drives tumor immune escape and poor outcomes (33). Although the immune system can eliminate tumor cells through the “cancer immune cycle” during tumor initiation and progression, malignant cells ultimately evade immune surveillance by establishing an immunosuppressive microenvironment (33, 34). Using the TIMER2.0 database, we analyzed the correlation between ZC3H15 expression and the immune-activating and immunosuppressive factors (**Figure 10**). The results indicated that ZC3H15 was positively associated with 14 immune-activating factors including CD276, CD80, ENTPD1, ICOS, IL6R, MICB, NT5E, PVR, TNFSF13, TNFSF13B, TNFSF15, TNFSF18, TNFSF4, and ULBP1 across over 20 tumor types (**Figure 10A**). Additionally, ZC3H15 showed a positive correlation with 4 immunosuppressive factors including CD160, CD274, KDR, and TGFBR1 in over 20 tumor types (**Figure 10B**). Collectively, ZC3H15 may exert complex regulatory effects on tumor immunity.

**Figure 10:**
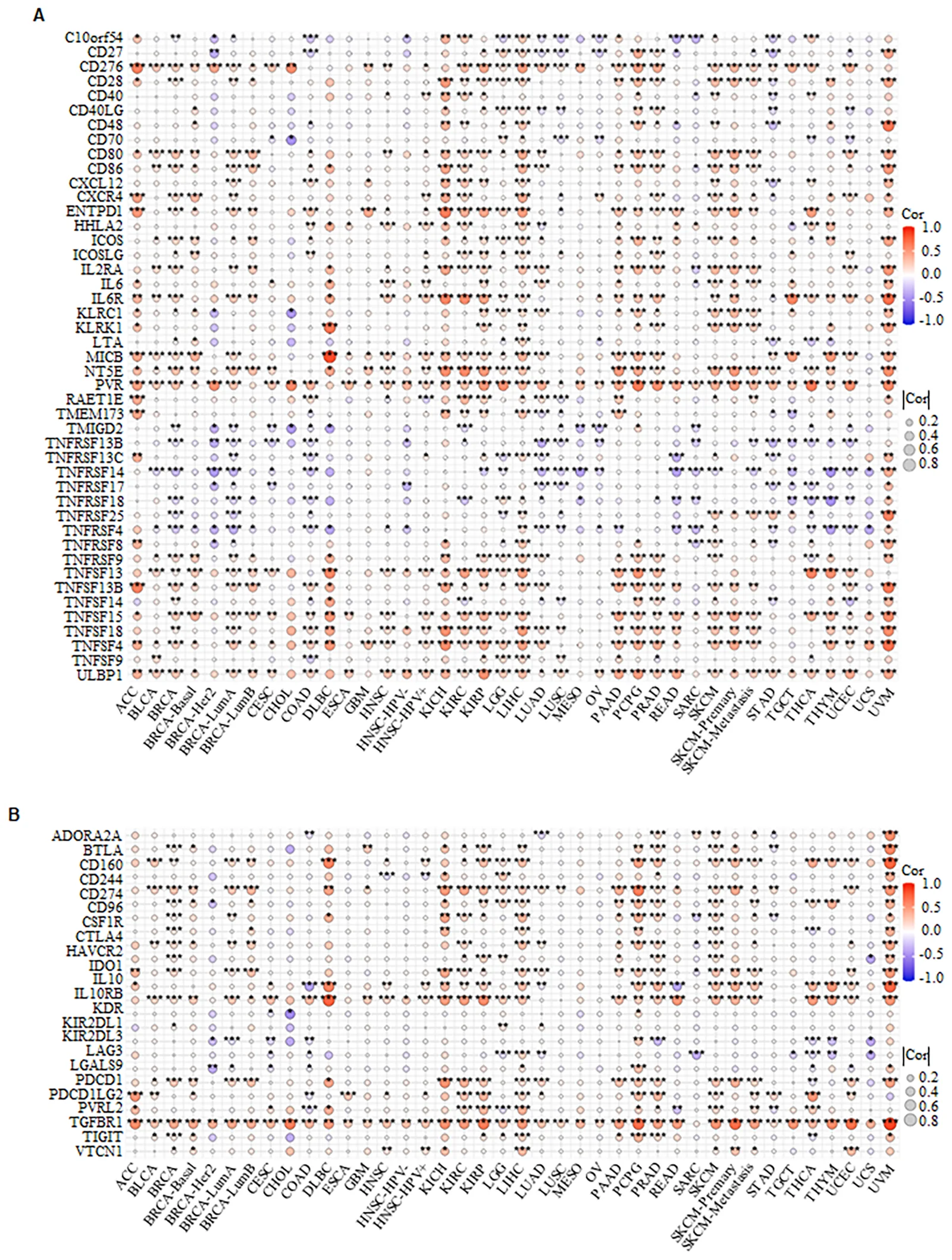
Correlation of ZC3H15 expression with Immunostimulators **(A)** and Immunoinhibitors **(B)** in pan-cancer. *adj. P < 0.05, **adj. P < 0.01, ***adj. P < 0.001.

### GSEA analysis of potential functions and pathway of ZC3H15 in human cancers

We found that ZC3H15 was significantly upregulated in five types of cancer (namely LIHC, LUAD, BRCA, LUSC, and HNSC) and was strongly associated with the malignant progression of these cancers as evidenced by gene expression, protein expression, staging, OS, PFI, and DSS. We validated the expression of ZC3H15 using the GEO database and found that ZC3H15 expression was significantly elevated in all five cancer types compared with matched normal tissues (**Figures 11A–E**).

**Figure 11:**
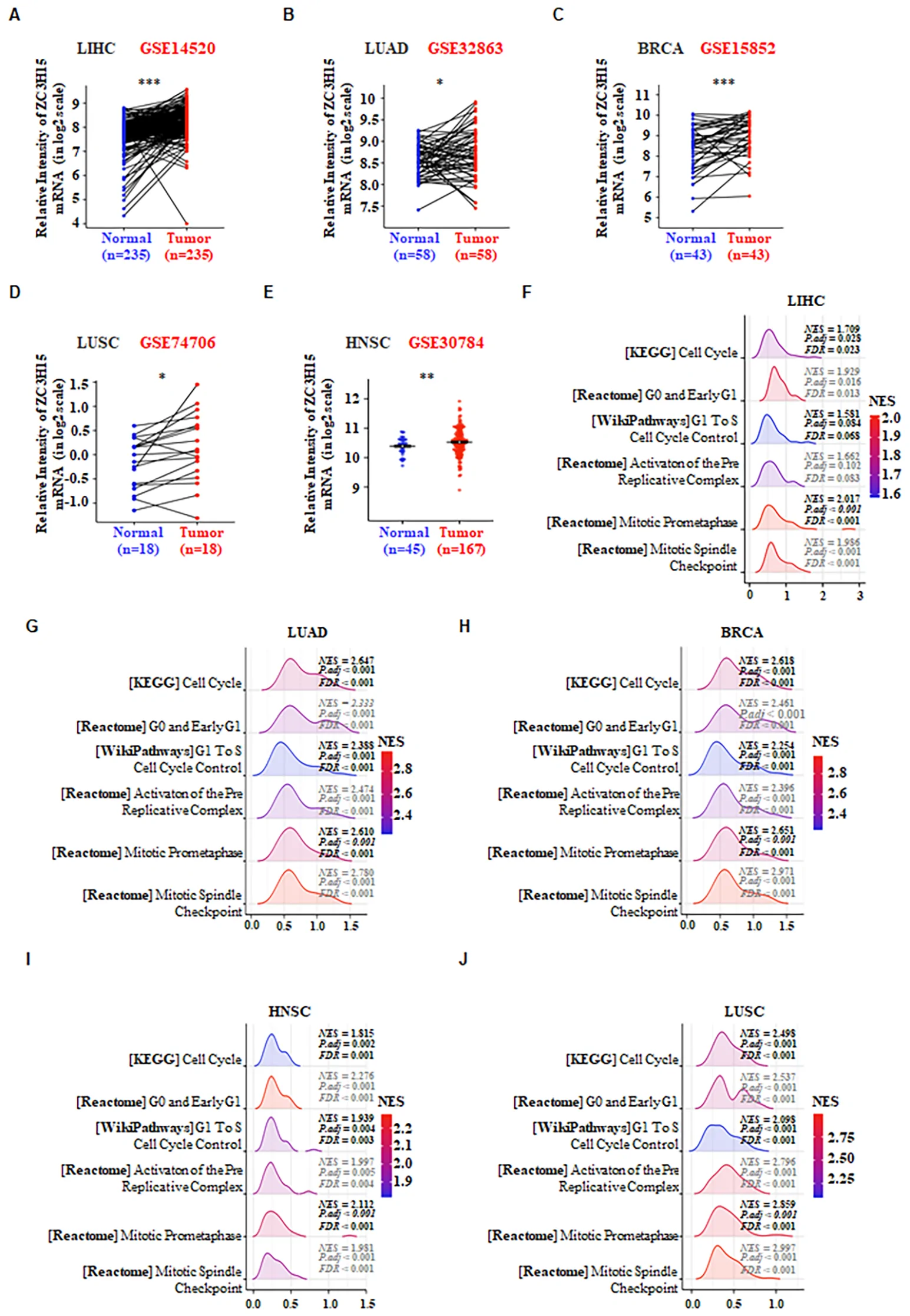
Functional enrichment analysis of ZC3H15 in pan-cancer. **(A)** A paired box plot illustrating the differential expression of ZC3H15 between LIHC tumors and adjacent normal tissues from the GSE14520 dataset. **(B)** A paired box plot illustrating the differential expression of ZC3H15 between LUAD tumor tissue and paired adjacent normal tissue from the GSE32863 dataset. **(C)** A paired box plot illustrating the differential expression of ZC3H15 between BRCA tumor tissue and adjacent normal tissue from the GSE15852 dataset. **(D)** A paired box plot illustrating the differential expression of ZC3H15 between LUSC tumor tissue and adjacent normal tissue from the GSE74706 dataset. **(E)** A box plot illustrating the differential expression of ZC3H15 between HNSC tumor tissues and normal tissues from the GSE30784 dataset. **(F–J)** Ridge plots displaying GSEA results of differentially expressed genes between ZC3H15-high and ZC3H15-low patients in LIHC **(F)**, LUAD **(G)**, BRCA **(H)**, HNSC **(I)**, and LUSC **(J)** cancer types.

To elucidate the mechanism by which ZC3H15 drives tumorigenesis, we conducted GSEA. We performed GSEA on these five cancer types: LIHC, LUAD, BRCA, LUSC, and HNSC (**Figures 11F–J**). This analysis identified differentially expressed genes between patients with high and low levels of ZC3H15 in LIHC, LUAD, BRCA, and LUSC, and HNSC. To better elucidate the mechanism by which ZC3H15 promotes tumor progression, we analyzed the pathways enriched in all five types of tumors mentioned above (**Figures 11F–J**). The shared pathways were mainly enriched in cell cycle-related signaling pathways. For example, multiple cell cycle-related pathways were activated in ZC3H15-high patients, including G1/S phase related signaling pathways, DNA replication related pathways, and mitosis-related pathways (**Figures 11F–J**). Interestingly, our results demonstrated that several mitosis-related pathways were also activated, including spindle formation, formation and assembly of the cell division center (centrosome), sister chromosome separation, and cytoplasmic division at the end of mitosis (**Supplementary Figure S6A**). These processes are closely linked to the dynamic regulation of the cytoskeleton. GSEA in this study showed that multiple cytoskeleton-related pathways were activated in samples with high ZC3H15 expression. Examples include recruitment of NuMA to mitotic centrosomes pathway, Rho-GTPases activated formins, Aurora B pathway, and Polo-like kinase 1 (PLK1) pathway (**Supplementary Figure S6A**).

GO and KEGG analyses further supported the role of ZC3H15 in cancer. To further examine the functions of ZC3H15 in cancers, we obtained ZC3H15-related genes via the Gene Expression Profiling Interactive Analysis (GEPIA) database and performed GO function and KEGG pathway enrichment analyses on these genes. To investigate how ZC3H15 promotes tumorigenesis, we used the GEPIA database to identify the top 100 genes with expression patterns similar to ZC3H15 in all tumor types. GO term analysis suggested that ZC3H15 may be involved in tumorigenesis through biological processes such as “mRNA processing”, “ribonucleoprotein complex biogenesis”, “translational initiation”, “DNA replication”, “chromosome segregation”, “sister chromosomal segregation”, “mitotic spindle organization”, “positive regulation of Arp2/3 complex-mediated actin nucleation”, and “mitotic nuclear division” (**Supplementary Figure S6B**). KEGG pathway enrichment analysis revealed that these ZC3H15-associated genes were primarily enriched in pathways including “Spliceosome”, “Ribosome biogenesis in eukaryotes”, and “Cell cycle” (**Supplementary Figure S6B**).

In summary, ZC3H15 may primarily influence tumor progression by regulating multiple processes such as DNA replication, mitosis, and the tumor immune microenvironment (**Figures 8**–**11**; **Supplementary Figure S6**).

### ZC3H15 knockdown inhibits proliferation, migration, and invasion of hepatocellular carcinoma and breast cancer cells *in Vitro*

Following the synthesis of ZC3H15-specific siRNAs, ZC3H15 was knocked down in hepatoma cells. Results indicated that all three siRNA sequences effectively reduced ZC3H15 levels (**Figure 12A**). CCK-8 assays demonstrated that ZC3H15 knockdown significantly inhibited the proliferation of hepatoma cells (**Figure 12B**). GSEA implicated ZC3H15 in regulating the cytoskeleton **Supplementary Figure S6**). Therefore, we performed Transwell assays to evaluate cell migration and invasion upon ZC3H15 knockdown (**Figures 12C, D**). The results indicated that ZC3H15 knockdown significantly inhibited the migration and invasion capabilities of liver cancer cells (**Figures 12C, D**, respectively).

**Figure 12:**
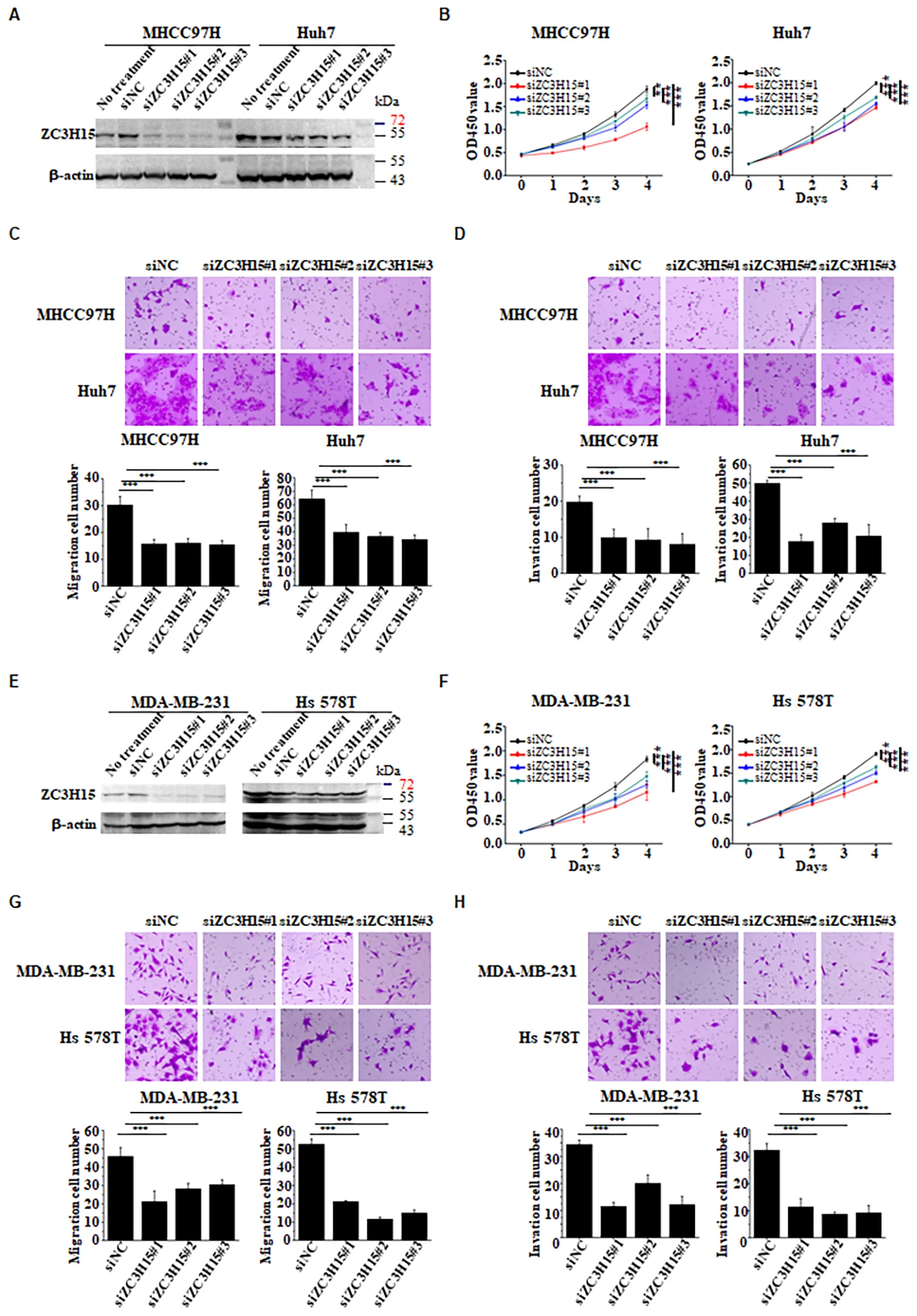
Following ZC3H15 knockdown, the proliferation, migration, and invasion of MHCC97H and Huh7 hepatoma cells, as well as MDA-MB-231 and Hs 578T breast cancer cells, were significantly inhibited. **(A)** Knockdown of ZC3H15 in MHCC97H and Huh7 cells using siRNAs (si-ZC3H15 #1, #2, and #3) was validated by Western blot analysis. β-actin served as a loading control. **(B)** After suppressing ZC3H15 expression with siRNAs (si-ZC3H15 #1, #2, and #3) in MHCC97H and Huh7 cells, the impact on cell viability was assessed using the CCK-8 assay. The migration **(C)** and invasion **(D)** of MHCC97H and Huh7 liver cancer cells were evaluated using Transwell assays following the inhibition of ZC3H15 expression with siRNA (si-ZC3H15 # 1, # 2, and # 3). Data are presented as mean ± SD (n = 6). **(E)** Knockdown of ZC3H15 in MDA-MB-231 and Hs 578T breast cancer cells using siRNAs (si-ZC3H15 #1, #2, and #3) was validated by Western blot analysis. β-actin served as a loading control. **(F)** After suppressing ZC3H15 expression with siRNAs (si-ZC3H15 #1, #2, and #3) in MDA-MB-231 and Hs 578T breast cancer cells, the impact on cell viability was assessed using the CCK-8 assay. The migration **(G)** and invasion **(H)** of MDA-MB-231 and Hs 578T breast cancer cells were evaluated using Transwell assays following the inhibition of ZC3H15 expression with siRNA (si-ZC3H15 # 1, # 2, and # 3). Data are presented as mean ± SD (n = 6). *P < 0.05, **P < 0.01, ***P < 0.001.

To bolster the evidence for ZC3H15’s pan-cancer role in tumorigenesis, we performed additional experimental validation using two breast cancer cell lines. Transfection with three distinct ZC3H15-specific siRNAs significantly reduced ZC3H15 expression in the MDA-MB-231 and Hs 578T breast cancer cell lines (**Figure 12E**). CCK-8 assays revealed that ZC3H15 knockdown significantly inhibited breast cancer cell proliferation (**Figure 12F**). Transwell assays demonstrated that ZC3H15 knockdown significantly attenuated breast cancer cell migration (**Figure 12G**) and invasion (**Figure 12H**).

GSEA suggested that ZC3H15 was closely associated with cell cycle regulation (**Figures 11F–J**). Flow cytometry analysis revealed that after knocking down ZC3H15, hepatoma cells were arrested in the G2/M phase (**Supplementary Figures S7A, B**). To elucidate the mechanism underlying G2/M phase arrest, we initially employed an EdU assay, which revealed no significant reduction in DNA replication in hepatoma cells following ZC3H15 knockdown (**Supplementary Figures S7C–E**). Immunofluorescence staining was performed using mitosis-specific pH3 antibodies and α-tubulin. The results showed that following ZC3H15 knockdown, the proportion of pH3-positive hepatoma cells decreased, indicating reduced mitotic activity (**Figures 13A, B, D, E**). Furthermore, we quantitatively analyzed the distribution of different mitotic phases in hepatoma cells following ZC3H15 knockdown. The main characteristic of early mitosis is the formation of spindle fibers visualized using α-tubulin antibodies, while chromosomes visualized using pH3 antibodies are dispersed in the center of the spindle (35). Statistical analysis revealed that the proportion of hepatoma cells in early mitosis following ZC3H15 knockdown was significantly higher than that in the control group (**Figures 13A, C, D, F**). These findings suggest that ZC3H15 may not be involved in DNA replication, but rather affect the early stages of mitosis.

**Figure 13:**
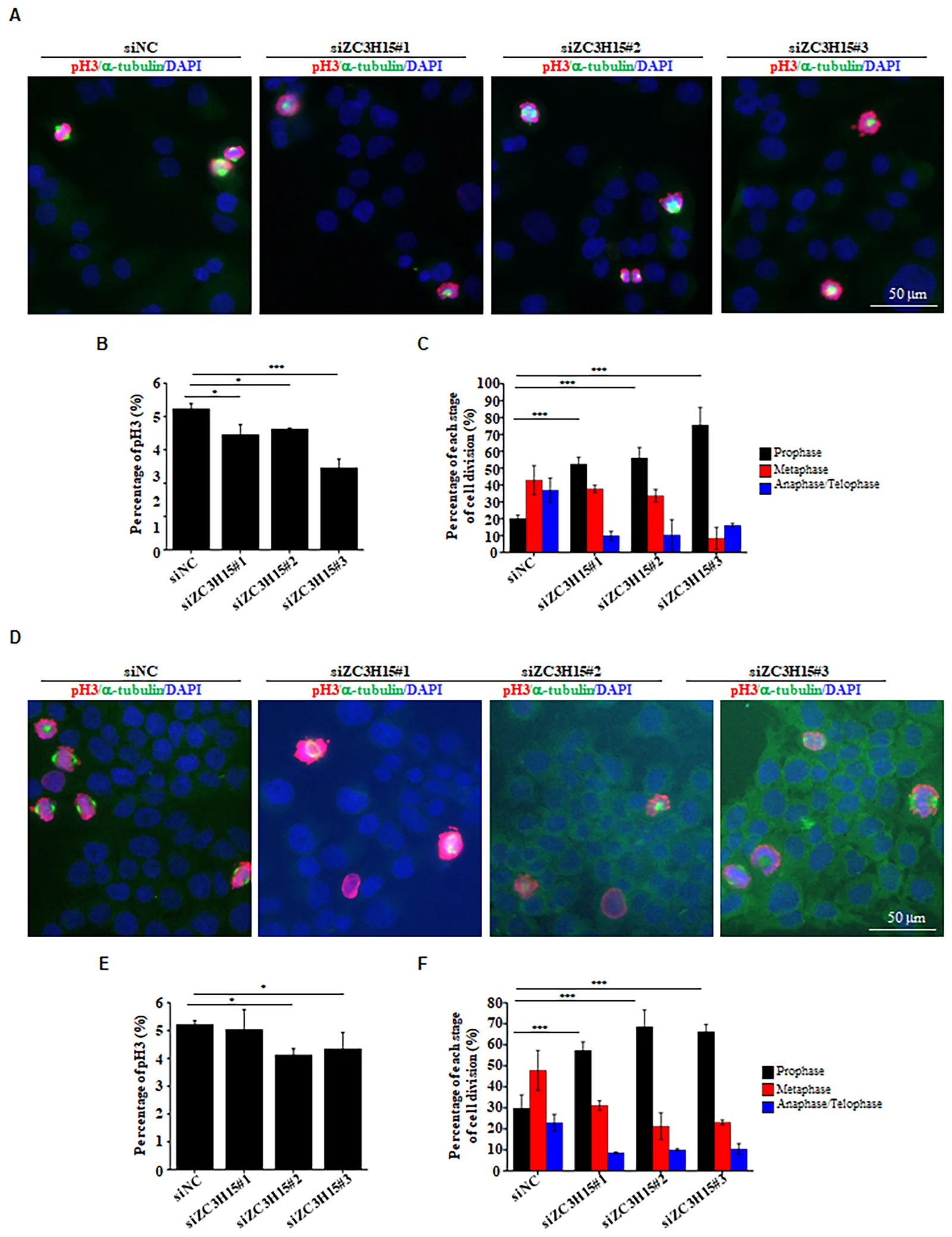
Following ZC3H15 knockdown, prometaphase arrest was observed in MHCC97H and Huh7 hepatoma cells. **(A)** Representative images depicting double immunostaining for α-tubulin and pH3 in MHCC97H cells treated with siRNAs targeting ZC3H15 for 3 days. **(B)** The percentage of cells with pH3-positive staining in MHCC97H cells was quantified, and data are presented as mean ± standard deviation (n = 3 biological replicates). **(C)** Quantification of MHCC97H cells across different stages of mitosis following ZC3H15 knockdown via double immunostaining for α-tubulin and pH3. **(D)** Representative images depicting double immunostaining for α-tubulin and pH3 in Huh7 cells treated with siRNAs targeting ZC3H15 for 3 days. **(E)** The percentage of cells with pH3-positive staining in Huh7 cells was quantified, and data are presented as mean ± standard deviation (n = 3 biological replicates). **(F)** Quantification of Huh7 cells across different stages of mitosis following ZC3H15 knockdown via double immunostaining for α-tubulin and pH3. *P < 0.05, **P < 0.01, ***P < 0.001.

The core of cell cycle regulation is the synergistic effect of cyclins and cyclin-dependent kinases (CDKs), which drive the orderly completion of various stages of cell division through precise temporal expression and activation (36). We first analyzed the correlation between ZC3H15 and cyclin protein levels in LIHC, HNSC, BRCA, LUAD, and LUSC. The results showed that only CCNB1 was positively correlated with ZC3H15 in all five cancers (**Supplementary Figure S8A**). CCNB1 is a cyclin that plays a crucial role in the G2/M transition (37). It primarily binds to CDK1 to form maturation-promoting factor (MPF), which initiates mitosis (38). The inhibition or downregulation of cyclin B/CDK1 complex activity may induce cell cycle arrest in the G2/M phase (38). Therefore, we further examined the correlation between CDK1 and ZC3H15 protein levels. The results showed that CDK1 was positively correlated with ZC3H15 in all five of these cancers (**Supplementary Figure S8B-G**).

Wu et al. recently demonstrated that, in non-small cell lung cancer (NSCLC), ZC3H15 promotes PTEN ubiquitination via TRIM56, which subsequently activates the AKT/mTOR pathway (39). To investigate whether ZC3H15 promotes liver cancer progression by activating the AKT/mTOR pathway, we conducted Western blot analysis. The results demonstrated that ZC3H15 significantly activated this pathway in Huh7 cells (**Supplementary Figure S8H**). Conversely, ZC3H15 did not activate the AKT/mTOR pathway in MHCC97H cells (**Supplementary Figure S8H**).

Taken together, ZC3H15 may promote tumor cell proliferation by modulating cell cycle proteins. Furthermore, through the modulation of cytoskeletal dynamics, ZC3H15 may also promote tumor cell migration and invasion.

## Discussion

Pan-cancer research is widely used to identify tumor molecular markers and signaling pathways (5). This approach aims to gain a more comprehensive and in-depth understanding of the molecular mechanisms underlying tumor occurrence and development. Pan-cancer analysis integrates data from multiple cancer types to analyze mRNA and protein expression levels across different tumors. It examines their correlations with prognosis, the tumor microenvironment, and regulatory mechanisms. This approach helps to identify similarities and differences among tumor types, discover common characteristics across cancers, and provide new insights into tumor prevention and personalized treatment strategies (5, 40). These findings can aid in early diagnosis and the formulation of effective treatment strategies. ZC3H15 is highly expressed in several tumor types, including melanoma, gastric cancer, liver cancer, glioblastoma, and acute myeloid leukemia (10–14). Its elevated expression correlates with poor patient prognosis, indicating a potential oncogenic role and suggesting that ZC3H15 may serve as a biomarker for cancer prognosis (10–14). However, the molecular mechanisms mediated by ZC3H15 in different cancer types remain unclear and require further investigation. This study aims to comprehensively examine ZC3H15 expression in pan-cancer, to reveal ZC3H15’s commonalities, differences, and new associations across various malignancies. Our findings indicated that ZC3H15 played an important role in the progression and prognosis of many tumors.

The HPA dataset showed widespread expression of ZC3H15 mRNA in normal tissues (**Figure 1A**). Data from the TCGA and GTEx databases were subsequently used to compare ZC3H15 expression in 33 tumor types with that in healthy tissues. The results demonstrated that ZC3H15 expression was significantly upregulated in 17 different cancer types, including LIHC, LUAD, BRCA, HNSC, LUSC, READ, STAD, ESCA, COAD, CHOL, GBM, PAAD, THYM, DLBC, OV, LGG, and UCS (**Figures 1B, C**). The upregulation of ZC3H15 was particularly significant in LIHC, LUAD, BRCA, LUSC, and HNSC. Moreover, its association with pathological stages varied among different cancer types (**Figures 1**, **2**; **Supplementary Figure S1**). These results indicated that ZC3H15 may play a significant role in tumor development, potentially influencing tumor growth and progression. In addition, studies on protein expression levels using the CPTAC database showed that ZC3H15 protein was significantly upregulated in LIHC, LUAD, LUSC, BRCA, HNSC, COAD, GBM, UCEC, and OV (**Figures 5**, **6**). Immunohistochemistry data from the HPA database showed that many cancer tissue types, including LIHC, LUAD, LUSC, BRCA, and HNSC, exhibited moderate to strong staining intensity compared to normal tissues, indicating higher ZC3H15 expression levels. This finding supported an association between ZC3H15 expression and the clinicopathological characteristics of various malignant tumors.

Our pan-cancer analysis results indicated that ZC3H15 was highly expressed not only in previously reported tumors such as liver cancer, glioblastoma, and gastric cancer, but also in other cancer types such as LUAD, BRCA, HNSC, LUSC, READ, ESCA, COAD, and CHOL. It is worth noting that the expression data of ZC3H15 in the TCGA database may not be consistent with the data results of TCGA+GTEx. For instance, within the TCGA database alone, ZC3H15 mRNA expression in GBM showed no significant increase compared to normal tissues (**Figure 1B**); however, analysis of the comprehensive TCGA and GTEx datasets revealed a significant upregulation in GBM (**Figure 1C**). The primary cause of this discrepancy is likely the small sample size of normal tissues in the TCGA database (n=5). Similar discrepancies observed in PAAD and THYM between the standalone TCGA database and the combined TCGA-GTEx dataset may also be attributed to these factors.

In addition, to evaluate the prognostic and diagnostic value of ZC3H15, Kaplan-Meier survival curves and ROC curves were used for analysis. Kaplan-Meier survival analysis showed a significant correlation between elevated ZC3H15 levels and adverse clinical outcomes across multiple cancer types. Univariate Cox regression analysis indicated that ZC3H15 was a risk factor for poor prognosis in patients with LIHC, LUAD, BRCA, PAAD, LGG, CESC, UCEC, PRAD, MESO, KIRP, KICH, and ACC. The ROC curves showed that ZC3H15 had good predictive accuracy for LIHC, LUAD, LUSC, STAD, ESCA, COAD, BRCA, HNSC, READ, KICH, GBM, and THCA (AUC > 0.7). These findings highlighted the significant prognostic and diagnostic potential of ZC3H15 in the cancers mentioned above, indicating that it may become a promising biomarker or therapeutic target for cancer.

The tumor microenvironment, which supports the growth and survival of cancer cells, constitutes a highly complex ecosystem composed of cancer cells, vasculature, peripheral immune and inflammatory cells, and other cellular components (33, 34). Stromal and immune cells in the tumor microenvironment can influence cancer prognosis and patient survival (41). The interaction of cancer cells with the tumor microenvironment is a key factor influencing tumor development, prognosis, and treatment response (33). Our analysis revealed a negative correlation between ZC3H15 expression and the presence of stromal and immune cells in the tumor microenvironment.

Although no studies have directly investigated the association between ZC3H15 expression and tumor-infiltrating immune cells, existing studies indicate that ZC3H15 is involved in immune responses (15, 42). For example, in a mouse model of bone cancer pain, ZC3H15 expression is significantly upregulated and is associated with severe oxidative stress, inflammatory responses, and microglial activation (15). We evaluated the correlation between ZC3H15 expression and tumor-infiltrating immune cells using seven algorithms, including MCPcounter and TIMER. Our analysis of immune cells and subpopulations infiltrating into the tumor associated with ZC3H15 expression yielded complex and sometimes contradictory results. Although these algorithms are widely used to evaluate immune infiltration, their distinct underlying methodologies result in divergent outcomes.

Nevertheless, consistent findings were also observed across these algorithms. For example, results from the MCPcounter, TIMER, and quanTIseq algorithms indicated that ZC3H15 was positively associated with tumor infiltration of neutrophils in many types of tumors (**Figures 8B–D**). Results from the MCPcounter algorithm indicated that, excluding COAD, ESCA, LUAD, READ, and STAD, ZC3H15 was positively correlated with neutrophil infiltration across various tumor types. Specifically, moderate-to-strong positive correlations were observed in ACC, BLCA, BRCA-Her2, BRCA-LumA, DLBC, HNSC-HPV+, KICH, KIRC, KIRP, LGG, MESO, PAAD, PCPG, PRAD, SKCM, SKCM-Metastasis, SKCM-Primary, TGCT, THCA, THYM, UCEC, and UVM. TIMER analysis indicated that, except for COAD, ESCA, GBM, HNSC, HNSC-HPV-, LGG, LUSC, SARC, STAD, and TGCT, ZC3H15 was positively correlated with neutrophil infiltration. Among these, moderate-to-strong positive correlations were observed in BRCA, BRCA-LumA, BRCA-LumB, CHOL, DLBC, KICH, KIRC, PAAD, PCPG, PRAD, SKCM, SKCM-Metastasis, SKCM-Primary, and THCA. quanTIseq analysis reveals that, excluding BRCA-Basal, CESC, ESCA, GBM, HNSC, HNSC-HPV+, HNSC-HPV-, LGG, LUAD, LUSC, SKCM, SKCM-Metastasis, and THYM, a positive correlation exists between ZC3H15 expression and neutrophil infiltration in UCS. Furthermore, moderate-to-strong positive correlations were noted in BRCA-Her2, KICH, KIRC, KIRP, MESO, THCA, and UVM. A study has found that in liver ischemia-reperfusion (IR) injury models, ZC3H15 expression is elevated, leading to the aggregation of CD11b+ macrophages and Ly6G+ neutrophils in the ischemic liver (42). The above results support the findings of this study, indicating that ZC3H15 may mediate neutrophil infiltration. Tumor-infiltrating neutrophils undergo reprogramming in the tumor microenvironment, exhibiting distinct phenotypes and functions that affect tumor development and treatment response (43, 44). For example, it has been reported that Ly6G+SiglecFhigh neutrophils in tumors have pro-tumor functions, promoting angiogenesis, immune suppression, and tumor invasion (45). Further experiments are needed to determine whether ZC3H15 directly mediates neutrophil infiltration, which neutrophil subtypes it influences in the tumor microenvironment, and how it affects tumor growth and development. Furthermore, MCPcounter and TIMER algorithms indicated that ZC3H15 may be positively correlated with macrophage tumor infiltration in various tumors (**Figures 8B, C**). Previous studies have reported that ZC3H15 can promote the activation of macrophages or stimulate the secretion of inflammatory factors by macrophages (15, 44). These reports support our findings. However, further experimental research is required to elucidate the mechanisms by which ZC3H15 modulates macrophage function within the tumor microenvironment. In addition, both ssGSEA and XCELL methods consistently demonstrated that in most cancer types, ZC3H15 was positively correlated with Th2 and negatively correlated with Th1 (**Figure 8E**; **Supplementary Figure S5B**). To confirm these findings, further experiments such as immunohistochemistry and flow cytometry are needed. Collectively, the significant correlations between ZC3H15 expression and various immune cell populations highlight its potential role in immune regulation.

ZC3H15 gene expression exhibited an overall negative correlation with estimated immune score, whereas it displayed a positive correlation with neutrophil and macrophage abundance in specific cancer types. While these findings seem paradoxical, this discrepancy was likely attributable to the interaction between cellular heterogeneity in the tumor microenvironment and unique properties of the tumor niche. The ESTIMATE immune score is derived from the aggregation of characteristic genes of immune cells in the transcriptome, encompassing major immune lineages such as T cells, B cells, and myeloid cells; therefore, it cannot be used as a quantitative indicator of specific immune cell types. When genes are highly expressed in tumor cells and stromal cells, the resulting high transcript abundance significantly elevates the overall gene expression level; however, this expression is unrelated to immune cell infiltration, thus diluting the overall correlation between gene expression and immune score. This dilution effect ultimately manifests as a negative correlation trend at the pan-cancer level. The composition of immune cells exhibits significant heterogeneity across different cancer types: some solid tumors are characterized by a microenvironment dominated by myeloid cells, in which the proportion of neutrophils and macrophages within the total immune cell population significantly exceeds that of T and B lymphocytes. The calculated immune score for this malignancy is primarily driven by the level of myeloid cell infiltration. In certain myeloproliferative cancers, ZC3H15 may directly upregulate chemokine expression and recruitment signals, particularly promoting the infiltration of circulating neutrophils and monocytes and macrophages into tumor tissue (**Figures 8**, **9**). Thus, ZC3H15 gene expression was positively correlated with the abundance of infiltrating cells in these two cell types. These results may also be related to the heterogeneity of cellular subpopulation functions, particularly the differences in macrophage polarization patterns and the functional subsets of neutrophils.

ZC3H15 is significantly upregulated in 17 types of cancer and has diagnostic and prognostic value for certain cancers. Existing literature indicates that ZC3H15, which is highly expressed in some malignant tumors, promotes tumor cell proliferation through multiple mechanisms. For example, in gastric cancer, ZC3H15 reduces the ubiquitination degradation of c-Myc protein by inhibiting the transcription of FBXW7, thereby stabilizing c-Myc protein and promoting cell proliferation (11). In GBM, ZC3H15 reduces EGFR ubiquitination and enhances the stability of EGFR protein, activating the EGFR signaling pathway to promote the proliferation, migration, invasion, and tumorigenicity of GBM cells (12). In non-small cell lung cancer, ZC3H15 binds to PTEN through its DFRP domain and recruits E3 ligase TRIM56 to promote ubiquitin-mediated degradation of PTEN, thereby activating the downstream AKT-mTOR signaling pathway, which is one of the core mechanisms driving cell proliferation and inhibiting apoptosis (39). Does ZC3H15 drive the initiation and progression of diverse malignancies through a common mechanism?

Therefore, in this study, we aimed to elucidate the common mechanisms underlying ZC3H15-promoted initiation and progression of various cancers via a pan-cancer analysis. We focused on studying five types: LIHC, LUAD, BRCA, LUSC, and HNSC. Then, we employed two complementary enrichment methods to analyze these cancers and further confirmed the mechanism of ZC3H15 promoting cancer through experiments. GSEA indicated that ZC3H15 may be involved in cancer initiation and progression by regulating cell cycle events, including DNA replication and mitosis (**Figures 11F–J**). Genes co-expressed with ZC3H15 were extracted from the GEPIA database and subjected to GO and KEGG pathway analysis (**Supplementary Figure S6B**). The results of the GO and KEGG pathway analyses corroborated the findings from the GSEA. To clarify which cell cycle events ZC3H15 primarily regulated during cancer development, we knocked down ZC3H15 in two liver cancer cell lines. CCK-8 assays revealed that ZC3H15 knockdown significantly inhibited cell proliferation and induced cell cycle arrest at the G2/M phase (**Supplementary Figure S7A**). Subsequent immunofluorescence staining revealed that after ZC3H15 knockdown, liver cancer cells predominantly accumulated in prometaphase (**Figure 13**). Previous studies have shown that inhibiting ZC3H15 in melanoma cells reduces cell proliferation and induces cell cycle arrest in the G0/G1 phase (14). However, we demonstrated for the first time that ZC3H15 promotes cancer cell proliferation by regulating mitotic prometaphase, representing a novel mechanism of ZC3H15-mediated oncogenesis.

Our results demonstrated that ZC3H15 was involved in several key processes during mitosis, including spindle formation, centrosome assembly and maturation, sister chromosome separation and cytokinesis (**Supplementary Figure S6A**). Multiple pathways involved in regulating centrosome movement and chromosome segregation-such as the “recruitment of NuMA_to mitotic centrosomes pathway” were significantly enriched in patients with high ZC3H15 expression. Moreover, the pathways “Rho-GTPase-activated-formins,” “Cilium assembly,” and “Positive regulation of Arp2/3 complex-mediated actin nucleation” were also enriched in patients with high ZC3H15 expression. We speculated that ZC3H15 may regulate the cytoskeleton to participate in key mitotic events. Although no studies have directly reported the involvement of ZC3H15 in cytoskeletal regulation, developmentally regulated GTP-binding protein 1 (DRG1), which binds to ZC3H15, is involved in the regulation of microtubules (9, 46). Previous studies have shown that DRG1 promotes microtubule aggregation, bundling, and stabilization (46). These processes influence spindle formation during cell division. The absence of DRG1 delays the progression of HeLa cells from early to late mitotic stages by slowing down spindle assembly (46). Based on the interaction between DRG1 and ZC3H15, it is plausible that ZC3H15 may also participate in cytoskeletal regulation. However, whether ZC3H15 truly participates in these processes requires further experimental confirmation.

The CDK1/cyclin B1 complex plays a crucial role in promoting the G2/M transition (37). Abnormal CDK1/cyclin B1 signaling can lead to prolonged mitotic prophase (38). We found through protein databases that ZC3H15 was positively correlated with CDK1 and cyclin B1 (**Supplementary Figure S8A**). These results suggested that ZC3H15 knockdown may induce prometaphase arrest by disrupting the function of the CDK1/cyclin B1 complex. The CDK1/cyclin B1 complex is regulated hierarchically and precisely. CDK1 is overexpressed in various tumors and participates in the process of tumor cell proliferation. CDK1 (cyclin-dependent kinase 1) is a core kinase that regulates the transition of eukaryotic cells from G2 phase to M phase (mitotic phase). CDK1 itself has no kinase activity and must bind to Cyclin B to form MPF (Maturation Promoting Factor or M-phase Promoting Factor) in order to be activated. The activity of CDK1 is also regulated by the phosphorylation status of different sites. For example, Wee1 kinase phosphorylates CDK1 at the Thr14 and Tyr15 sites, rendering it inactive (47). Phosphorylation by CAK (CDK-activating kinase) at the Thr161 residue is essential for full activation (48). Phosphatase Cdc25 removes phosphate groups from Thr14/Tyr15, fully activating CDK1 (37). In addition, Cyclin B is ubiquitinated in the late M phase and degraded by proteasomes, leading to inactivation of CDK1 and promoting cell exit from mitosis (49). However, how ZC3H15 regulates the CDK1/cyclin B1 complex requires further investigation in our future research.

siRNA technology faces two primary limitations of off-target effects and the transient nature of gene silencing. These limitations can directly compromise the integrity of experimental data. Off-target effects pose a significant challenge in siRNA technology, as they can lead to the unexpected silencing of non-target genes. This results in phenotypic changes attributable not only to the knockdown of target genes but also to off-target effects, thereby complicating data interpretation and potentially yielding false-positive results. Using multiple siRNAs targeting the same gene not only enhances silencing efficiency but also reduces off-target effects by lowering the concentration of any single siRNA. Therefore, we employed three siRNAs targeting distinct ZC3H15 sites to evaluate the efficacy of ZC3H15 knockdown. In addition, siRNA-mediated silencing is inherently transient due to its limited duration. The silencing effect typically lasts approximately one week. Therefore, all experimental assessments were conducted within this 7-day period in this study.

In this study, we analyzed the diagnostic and prognostic value, genetic alterations, DNA methylation status, and tumor-infiltrating immune cells of ZC3H15. Based on these comprehensive analyses, ZC3H15 could serve as a biomarker for certain types of cancer. Moreover, the pathway enrichment analysis and subsequent liver cancer cell experiments demonstrated that ZC3H15 primarily promotes tumor progression by regulating the early stages of mitosis. However, this study has several limitations. First, our data were obtained from different databases, which may introduce selection bias. Second, the relationship between ZC3H15 and tumor immune cell infiltration in this study relies primarily on bioinformatics analysis and prediction without adequate experimental validation. In summary, our findings highlight the potential role of ZC3H15 in tumor progression, particularly its capacity to promote tumor cell proliferation and modulate the tumor microenvironment, and elucidate the mechanisms underlying ZC3H15-driven carcinogenesis via pathway enrichment analysis and experimental validation.

## Conclusions

ZC3H15 expression correlated with several clinical features in multiple malignant tumors. In some cancers, higher expression predicted poorer OS, DSS, and PFI, and was also associated with the infiltration of various immune cells. Knockdown of ZC3H15 suppressed cell proliferation, migration, and invasion in the liver and breast cancer cell lines. The pathway enrichment analysis and follow-up hepatoma cell experiments demonstrated that ZC3H15 primarily promoted tumor progression by regulating the early stages of mitosis. This study suggests that ZC3H15 may be a valuable biomarker for tumor diagnosis, immune cell infiltration, and prognosis.

## Data Availability

The original contributions presented in the study are included in the article/**Supplementary Material**. Further inquiries can be directed to the corresponding authors.

## Glossary

aDC: activated DC
ADORA2B: adenosine receptor A2B
AFP: Alpha-fetoprotein
AUC: area under the curve
BCP: bone cancer pain
CBL: Casitas B-lineage
CI: confidence interval
CNVs: copy number variations
CPTAC: Clinical Proteomic Tumor Analysis Consortium
DCs: dendritic cells
DE: differentially expressed genes
DRG1: developmentally regulated GTP-binding protein 1
DSS: disease-specific survival
EdU: 5-ethynyl-2’-deoxyuridine
EGFR: epidermal growth factor receptor
FBXW7: F-box and WD repeat domain containing seven
FDR: false discovery rate
GEO: Gene Expression Omnibus
GEPIA: Gene Expression Profiling Interactive Analysis
GO: Gene Ontology
GSCA: Gene Set Cancer Analysis
GSEA: gene set enrichment analysis
GTEx: Genotype-Tissue Expression
HPA: Human Protein Atlas
HR: hazard ratio
iDCs: immature dendritic cells
IκBα: nuclear factor kappa B inhibitor alpha
KEAP1: Kelch-like ECH-associated protein 1
KEGG: Kyoto Encyclopedia of Genes and Genomes
MAPK: mitogen-activated protein kinase
MCM: MiniChromosome Maintenance
MSI: microsatellite instability
NC cells: natural cytotoxicity cells
NES: Normalized Enrichment Score
NK cells: natural killer cells
NRF2: nuclear factor erythroid 2-related factor 2
OS: overall survival
pDCs: plasmacytoid dendritic cells
PFI: progression-free interval
PLK1: Polo-like kinase 1
Rb: retinoblastoma protein
ROC: receiver operating characteristic
SARS-CoV-2: severe acute respiratory syndrome coronavirus 2
ssGSEA: single-sample Gene Set Enrichment Analysis
TCGA: The Cancer Genome Atlas
Tcm: central memory T cells
Tem: T effector memory
TFH: T follicular helper
Tgd: gamma delta T cells
TLR: toll-like receptors
TMB: tumor mutation burden
TPM: transcripts per million
TRAF2: TNF receptor-associated factor 2
Treg: regulatory T cells
ZC3H15: Zinc finger CCCH-type protein 150.

## References

[1] Gilbertson RJ . Mapping cancer origins. Cell. (2011) 145:25–9. doi: 10.1016/j.cell.2011.03.019 21458665 PMC3077217

[2] Cordani M Dando I Ambrosini G González-Menéndez P . Signaling, cancer cell plasticity, and intratumor heterogeneity. Cell Commun Signal. (2024) 22:255. doi: 10.1186/s12964-024-01643-5 38702718 PMC11067149

[3] Finger AM Hendley AM Figueroa D Gonzalez H Weaver VM . Tissue mechanics in tumor heterogeneity and aggression. Trends Cancer. (2025) 11:806–24. doi: 10.1016/j.trecan.2025.04.004 40307158 PMC12350075

[4] Liao C Liu X Zhang C Zhang Q . Tumor hypoxia: From basic knowledge to therapeutic implications. Semin Cancer Biol. (2023) 88:172–86. doi: 10.1016/j.semcancer.2022.12.011 36603793 PMC9929926

[5] Chen F Wendl MC Wyczalkowski MA Bailey MH Li Y Ding L . Moving pan-cancer studies from basic research toward the clinic. Nat Cancer. (2021) 2:879–90. doi: 10.1038/s43018-021-00250-4 35121865

[6] Cen X Lan Y Zou J Chen R Hu C Tong Y . Pan-cancer analysis shapes the understanding of cancer biology and medicine. Cancer Commun (Lond). (2025) 45:728–46. doi: 10.1002/cac2.70008 40120098 PMC12328095

[7] Hitchen N Shahnam A Tie J . Circulating tumor DNA: A pan-cancer biomarker in solid tumors with prognostic and predictive value. Annu Rev Med. (2025) 76:207–23. doi: 10.1146/annurev-med-100223-090016 39570664

[8] McGrail DJ Pilié PG Rashid NU Voorwerk L Slagter M Kok M . High tumor mutation burden fails to predict immune checkpoint blockade response across all cancer types. Ann Onco. (2021) 32:661–72. doi: 10.1016/j.annonc.2021.02.006 33736924 PMC8053682

[9] Ishikawa K Azuma S Ikawa S Semba K Inoue J . Identification of DRG family regulatory proteins (DFRPs): specific regulation of DRG1 and DRG2. Genes Cells. (2005) 10:139–50. doi: 10.1111/j.1365-2443.2005.00825.x 15676025

[10] Jiang BG Wan ZH Huang J Li LM Liu H Fu SY . Elevated ZC3H15 increases HCC growth and predicts poor survival after surgical resection. Oncotarget. (2016) 7:37238–49. doi: 10.18632/oncotarget.9361 27191988 PMC5095072

[11] Hou J Huang P Lan C Geng S Xu M Liu Y . ZC3H15 promotes gastric cancer progression by targeting the FBXW7/c-Myc pathway. Cell Death Discov. (2022) 8:32. doi: 10.1038/s41420-022-00815-x 35064102 PMC8782901

[12] Hou J Xu M Gu H Pei D Liu Y Huang P . ZC3H15 promotes glioblastoma progression through regulating EGFR stability. Cell Death Dis. (2022) 13:55. doi: 10.1038/s41419-021-04496-9 35027542 PMC8758739

[13] Capalbo G Mueller-Kuller T Koschmieder S Klein HU Ottmann OG Hoelzer D . Characterization of ZC3H15 as a potential TRAF-2-interacting protein implicated in the NFkappaB pathway and overexpressed in AML. Int J Onco. (2013) 43:246–54. doi: 10.3892/ijo.2013.1924 23624947

[14] Li Q Hou J Guo C Zhang Y Liu L Hu H . ZC3H15 correlates with a poor prognosis and tumor progression in melanoma. BioMed Res Int. (2021) 2021:8305299. doi: 10.1155/2021/8305299 34988227 PMC8723872

[15] Huang LQ Yan TX Wang BS Li H Zhou NB . ZC3H15 suppression ameliorates bone cancer pain through inhibiting neuronal oxidative stress and microglial inflammation. Neoplasia. (2025) 61:101123. doi: 10.1016/j.neo.2025.101123 39908779 PMC11847137

[16] Wagner GP Kin K Lynch VJ . Measurement of mRNA abundance using RNA-seq data: RPKM measure is inconsistent among samples. Theory Biosci. (2012) 131:281–5. doi: 10.1007/s12064-012-0162-3 22872506

[17] Roessler S Jia HL Buchu A Forgues M Ye QH Lee JS . A unique metastasis gene signature enables prediction of tumor relapse in early-stage hepatocellular carcinoma patients. Cancer Res. (2010) 70:10202–12. doi: 10.1158/0008-5472.CAN-10-2607 21159642 PMC3064515

[18] Wu B Liu DA Guan L Myint PK Chin L Dang H . Stiff matrix induces exosome secretion to promote tumour growth. Nat Cell Biol. (2023) 25:415–24. doi: 10.1038/s41556-023-01092-1 36797475 PMC10351222

[19] Selamat SA Chung BS Girard L Zhang W Zhang Y Campan M . Genome-scale analysis of DNA methylation in lung adenocarcinoma and integration with mRNA expression. Genome Res. (2012) 22:1197–211. doi: 10.1101/gr.132662.111 22613842 PMC3396362

[20] Pau Ni IB Zakcria Z Muhammad R Abdullah N Ibrahim N Aina Emran N . Gene expression patterns distinguish breast carcinomas from normal breast tissues: the Malaysian context. Pathol Res Pract. (2010) 206:223–8. doi: 10.1016/j.prp.2009.11.006 20097481

[21] Marwitz S Depner S Dvornikov D Merkle R Szczygieł M Müller-Decker K . Downregulation of the TGFβ pseudoreceptor BAMBI in non-small cell lung cancer enhances TGFβ signaling and invasion. Cancer Res. (2016) 76:3785–801. doi: 10.1158/0008-5472.CAN-15-1326 27197161

[22] Chen C Méndez E Houck J Fan W Lohavanichbutr P Doody D . Gene expression profiling identifies genes predictive of oral squamous cell carcinoma. Cancer Epidemiol Biomarkers Prev. (2008) 17:2152–62. doi: 10.1158/1055-9965.EPI-07-2893 18669583 PMC2575803

[23] Thul PJ Lindskog C . The human protein atlas: A spatial map of the human proteome. Protein Sci. (2018) 27:233–44. doi: 10.1002/pro.3307 28940711 PMC5734309

[24] Chandrashekar DS Karthikeyan SK Korla PK Patel H Shovon AR Athar M . UALCAN: An update to the integrated cancer data analysis platform. Neoplasia. (2022) 25:18–27. doi: 10.1016/j.neo.2022.01.001 35078134 PMC8788199

[25] Gatsonis CA . Receiver operating characteristic analysis for the evaluation of diagnosis and prediction. Radiology. (2009) 253:593–6. doi: 10.1148/radiol.2533091633 19952022

[26] Cerami E Gao J Dogrusoz U Gross BE Sumer SO Aksoy BA . The cBio cancer genomics portal: an open platform for exploring multidimensional cancer genomics data. Cancer Discov. (2012) 2:401–4. doi: 10.1158/2159-8290.CD-12-0095 22588877 PMC3956037

[27] Liu CJ Hu FF Xie GY Miao YR Li XW Zeng Y . GSCA: an integrated platform for gene set cancer analysis at genomic, pharmacogenomic and immunogenomic levels. Brief Bioinform. (2023) 24:bbac558. doi: 10.1093/bib/bbac558 36549921

[28] Sun C Ma S Ccen Y Kim NH Kailas S Wang Y . Diagnostic value, prognostic value, and immune infiltration of LOX family members in liver cancer: Bioinformatic analysis. Front Oncol. (2022) 12:843880. doi: 10.3389/fonc.2022.843880 35311155 PMC8931681

[29] Hänzelmann S Castelo R Guinney J . GSVA: gene set variation analysis for microarray and RNA-seq data. BMC Bioinf. (2013) 14:7. doi: 10.1186/1471-2105-14-7 23323831 PMC3618321

[30] Li T Fu J Zeng Z Cohen D Li J Chen Q . TIMER2.0 for analysis of tumor-infiltrating immune cells. Nucleic Acids Res. (2020) 48:W509–14. doi: 10.1093/nar/gkaa407 32442275 PMC7319575

[31] Subramanian A Tamayo P Mootha VK Mukherjee S Ebert BL Gillette MA . Gene set enrichment analysis: a knowledge-based approach for interpreting genome-wide expression profiles. Proc Natl Acad Sci USA. (2005) 102:15545–50. doi: 10.1073/pnas.0506580102 16199517 PMC1239896

[32] Zhang C Liu W Yang P Lin R Pu L Zhang H . Dual roles of innate immune cells and cytokines in shaping the breast cancer microenvironment. Front Immunol. (2025) 16:1654947. doi: 10.3389/fimmu.2025.1654947 40909271 PMC12405171

[33] de Visser KE Joyce JA . The evolving tumor microenvironment: From cancer initiation to metastatic outgrowth. Cancer Cell. (2023) 41:374–403. doi: 10.1016/j.ccell.2023.02.016 36917948

[34] Barkley D Moncada R Pour M Liberman DA Dryg I Werba G . Cancer cell states recur across tumor types and form specific interactions with the tumor microenvironment. Nat Genet. (2022) 54:1192–201. doi: 10.1038/s41588-022-01141-9 35931863 PMC9886402

[35] Shao X Ding Z Zhou W Li Y Li Z Cui H . Intrinsic bioactivity of black phosphorus nanomaterials on mitotic centrosome destabilization through suppression of PLK1 kinase. Nat Nanotechnol. (2021) 16:1150–60. doi: 10.1038/s41565-021-00952-x 34354264

[36] Kapadia N Nurse P . Spatiotemporal orchestration of mitosis by cyclin-dependent kinase. Nature. (2025) 643:1391–9. doi: 10.1038/s41586-025-09172-y 40562936 PMC12310531

[37] Timofeev O Cizmecioglu O Settele F Kempf T Hoffmann I . Cdc25 phosphatases are required for timely assembly of CDK1-cyclin B at the G2/M transition. J Biol Chem. (2010) 285:16978–90. doi: 10.1074/jbc.M109.096552 20360007 PMC2878026

[38] Gobran M Politi AZ Welp L Jakobi J Urlaub H Lenart P . PLK1 inhibition delays mitotic entry revealing changes to the phosphoproteome of mammalian cells early in division. EMBO J. (2025) 44:1891–920. doi: 10.1038/s44318-025-00400-9 40033019 PMC11962124

[39] Wu P Yao P Zhao M Cheng M . ZC3H15 regulates the ubiquitination of PTEN via recruitment of TRIM56 and promotes malignant progression of non-small cell lung cancer. Cell Death Dis. (2026) 17:17. doi: 10.1038/s41419-025-08138-2 41513632 PMC12789496

[40] Connor AA Gallinger S . Pancreatic cancer evolution and heterogeneity: integrating omics and clinical data. Nat Rev Cancer. (2022) 22:131–42. doi: 10.1038/s41568-021-00418-1 34789870

[41] Ringquist R Ghoshal D Jain R Roy K . Understanding and improving cellular immunotherapies against cancer: From cell-manufacturing to tumor-immune models. Adv Drug Delivery Rev. (2021) 179:114003. doi: 10.1016/j.addr.2021.114003 34653533

[42] Qu X Yang T Wang X Xu D Yu Y Li J . Macrophage RIPK3 triggers inflammation and cell death via the XBP1–Foxo1 axis in liver ischaemia–reperfusion injury. JHEP Rep. (2023) 5:100879. doi: 10.1016/j.jhepr.2023.100879 37841640 PMC10568422

[43] Ugolini A De Leo A Yu X Scirocchi F Liu X Peixoto B . Functional reprogramming of neutrophils within the brain tumor microenvironment by hypoxia-driven histone lactylation. Cancer Discov. (2025) 15:1270–96. doi: 10.1158/2159-8290.CD-24-1056 40014923 PMC12133432

[44] Gao J Zhang W Li Q Zhao D Cai J Wang Q . Restricting lipid accumulation in tumor-infiltrating neutrophils mediates caloric restriction-induced anti-cancer effects. Cell Metab. (2026) 38:598–615.e7. doi: 10.1016/j.cmet.2025.11.007 41352341

[45] Pfirschke C Engblom C Gungabeesoon J Lin Y Rickelt S Zilionis R . Tumor-promoting Ly-6G+ SiglecFhigh cells are mature and long-lived neutrophils. Cell Rep. (2020) 32:108164. doi: 10.1016/j.celrep.2020.108164 32966785 PMC7508173

[46] Schellhaus AK Moreno-Andrés D Chugh M Yokoyama H Moschopoulou A De S . Developmentally regulated GTP binding protein 1 (DRG1) controls microtubule dynamics. Sci Rep. (2017) 7:9996. doi: 10.1038/s41598-017-10088-5 28855639 PMC5577222

[47] Josué Ruiz E Vilar M Nebreda AR . A two-step inactivation mechanism of Myt1 ensures CDK1/cyclin B activation and meiosis I entry. Curr Biol. (2010) 20:717–23. doi: 10.1016/j.cub.2010.02.050 20362450

[48] Fesquet D Labbé JC Derancourt J Capony JP Galas S Girard F . The MO15 gene encodes the catalytic subunit of a protein kinase that activates cdc2 and other cyclin-dependent kinases (CDKs) through phosphorylation of Thr161 and its homologues. EMBO J. (1993) 12:3111–21. doi: 10.1002/j.1460-2075.1993.tb05980.x 8344251 PMC413577

[49] Feng J Xie L Lu W Yu X Dong H Ma Y . Hyperactivation of p53 contributes to mitotic catastrophe in podocytes through regulation of the Wee1/CDK1/cyclin B1 axis. Ren Fail. (2024) 46:2365408. doi: 10.1080/0886022X.2024.2365408 38874119 PMC11182053

