# Supplementary material for: ZC3H15 in human tumors: a pan-cancer exploration of its clinical significance, molecular function, and association with the tumor immune microenvironment
Source: Front Immunol. 2026 Oct 5;17:1901516. doi: 10.3389/fimmu.2026.1901516 (PMC13677929; doi:10.3389/fimmu.2026.1901516)
Supplement: Supplementary file 1 [file Table1.docx]

Supplementary Material

# Supplementary Figures

**Supplementary Figure 1. Correlation between ZC3H15 expression levels and tumor metastasis status.** The relationship between ZC3H15 expression and clinical pathological grade I to IV in multiple cancer types, including LIHC (A), HNSC (B), KIRC (C), PAAD (D), PRAD (E), STAD (F). The relationship between ZC3H15 expression and tumor metastasis status in multiple cancer types, including LUAD (G), BRCA (H), LUSC (I), CHOL (J), KIRP (K). Statistical significance was indicated as follows: *P < 0.05, **P < 0.01, ***P < 0.001.

**Supplementary Figure 2. Validation and evaluation of nomograms.** (A) A nomogram to predict 1-year, 3-year, and 5-year OS in patients with LIHC. (B) A nomogram to predict 1-year, 3-year, and 5-year DSS in patients with LIHC. (C) A nomogram to predict 1-year, 3-year, and 5-year OS in patients with BRCA. (D) A nomogram to predict 1-year, 3-year, and 5-year PFI in patients with LUSC. (E) A nomogram to predict 1-year, 3-year, and 5-year PFI in patients with HNSC. (F) A nomogram to predict 1-year, 3-year, and 5-year DSS in patients with HNSC. (G) Calibration curves of the 1-year, 3-year, and 5-year OS nomogram for LIHC.(H) Calibration curves of the 1-year, 3-year, and 5-year DSS nomogram for LIHC. (I) Calibration curves of the 1-year, 3-year, and 5-year OS nomogram for BRCA. (J) Calibration curves of the 1-year, 3-year, and 5-year PFI nomogram for LUSC. (K) Calibration curves of the 1-year, 3-year, and 5-year PFI nomogram for HNSC. (L) Calibration curves of the 1-year, 3-year, and 5-year DSS nomogram for HNSC.

**Supplementary Figure 3. Detection of ZC3H15 protein expression levels and evaluation of its diagnostic value in cancer patients using the CPTAC dataset.** (A) KIRC, (B) COAD, (C) GBM, (D) PDAC, (E) UCEC, (F) OV. ∗P < 0.05，∗∗P < 0.01，∗∗∗P < 0.001.

**Supplementary Figure 4. Promoter methylation for ZC3H15 in various cancer types using the UALCAN dataset.** (A) HNSC, (B) LUSC, (C) KIRC, (D) UCEC, (E) PAAD, (F) BLCA, (G) PRAD, (H) COAD, (I) DLBC, (J) SKCM, (K) UVM. ∗P < 0.05，∗∗P < 0.01，∗∗∗P < 0.001.

**Supplementary Figure 5. Correlation of ZC3H15 expression with immune cells by three algorithm.** (A) ssGSEA algorithm, (B) CIBERSORT-ABS algorithm, (C)EPIC algorithm. aDC: activated DC, iDC: immature DC, pDC: Plasmacytoid DC, Tcm: T central memory, Tem: T effector memory, TFH: T follicular helper, Tgd: T gamma delta. ∗adj. P < 0.05，∗∗ adj. P < 0.01，∗∗∗ adj. P < 0.001.

**Supplementary Figure 6.** **Gene enrichment analysis of ZC3H15-related genes.** (A) Mitosis-related pathways were enriched in the ZC3H15-high patients compared to the ZC3H15-low patients in LIHC, LUAD, BRCA, LUSC, and HNSC as revealed by GSEA. (B) GO and KEGG analysis of ZC3H15-related genes.

**Supplementary Figure 7.** **Cell cycle and EdU results in ZC3H15-knockdown MHCC97H and Huh7 cells.** (A) The effect of ZC3H15 knockdown on the cell cycle of MHCC97H cells was assessed by flow cytometry. Representative images of the cell cycle are shown in the left panel. The quantitative analysis of the cell cycle is shown in the right panel. (B) The effect of ZC3H15 knockdown on the cell cycle of Huh7 cells was assessed by flow cytometry. Representative images of the cell cycle are shown in the left panel. The quantitative analysis of the cell cycle is shown in the right panel. (C) ZC3H15-knockdown MHCC97H cells were labeled with EdU to detect the number of cells undergoing active DNA synthesis. Representative images show MHCC97H cells labeled with EdU. (B) Quantification of MHCC97H cells following ZC3H15 knockdown, stained with EdU, relative to siNC. (D) Quantification of Huh7 cells following ZC3H15 knockdown, stained with EdU, relative to siNC. *: P＜0.05, **: P＜0.01, ***: P＜0.001, compared with siNC.

**Supplementary Figure 8.** **Analysis of the correlation between ZC3H15 and cell cycle-related genes obtained from protein databases across LIHC, LUAD, BRCA, HNSC, and LUSC cohorts.** (A) The correlations between ZC3H15 expression levels and the cell cycle proteins CCNA2, CCNB1, CCND1, CCNE1, and CCNE2 were analyzed, using protein data retrieved from the LIHC_HBV_protein, LUAD_APOLLO_protein, LUAD_CPTAC_protein, BRCA_BI_protein, HNSC_CPTAC_protein, and LUSC_CPTACAP_protein databases, respectively. (B-G) Correlation analysis between ZC3H15 expression levels and CDK1 expression levels using data retrieved from the LIHC_HBV_protein, LUAD_APOLLO_protein, LUAD_CPTAC_protein, BRCA_BI_protein, HNSC_CPTAC_protein, and LUSC_CPTACAP_protein databases, respectively. (H) Western blotting was performed to assess the activation of the AKT/mTOR pathway after ZC3H15 knockdown in MHCC97H and Huh7 hepatocellular carcinoma cell lines. ∗P < 0.05，∗∗P < 0.01，∗∗∗P < 0.001.

**Supplementary Figure 9. Original images of the Western blot results in Figure 12A.**

**Supplementary Figure 10. Original images of the Western blot results in Figure 12E.**

# Supplementary Tables

**Supplementary Table 1.** The full names of 33 cancer types.

| ACC | Adrenocortical carcinoma |
| --- | --- |
| BLCA | Bladder Urothelial Carcinoma |
| BRCA | Breast invasive carcinoma |
| CESC | Cervical squamous cell carcinoma and endocervical adenocarcinoma |
| CHOL | Cholangiocarcinoma |
| COAD | Colon adenocarcinoma |
| DLBC | Diffuse Large B-cell Lymphoma |
| ESCA | Esophageal carcinoma |
| GBM | Glioblastoma multiforme |
| HNSC | Head and Neck squamous cell carcinoma |
| KICH | Kidney Chromophobe |
| KIRC | Kidney renal clear cell carcinoma |
| KIRP | Kidney renal papillary cell carcinoma |
| LAML | Acute Myeloid Leukemia |
| LGG | Lower Grade Glioma |
| LIHC | Liver hepatocellular carcinoma |
| LUAD | Lung adenocarcinoma |
| LUSC | Lung squamous cell carcinoma |
| MESO | Mesothelioma |
| OV | Ovarian serous cystadenocarcinoma |
| PAAD | Pancreatic adenocarcinoma |
| PCPG | Pheochromocytoma and Paraganglioma |
| PRAD | Prostate adenocarcinoma |
| READ | Rectum adenocarcinoma |
| SARC | Sarcoma |
| SKCM | Skin Cutaneous Melanoma |
| STAD | Stomach adenocarcinoma |
| TGCT | Testicular Germ Cell Tumors |
| THCA | Thyroid carcinoma |
| THYM | Thymoma |
| UCEC | Uterine Corpus Endometrial Carcinoma |
| UCS | Uterine Carcinosarcoma |
| UVM | Uveal Melanoma |

**Supplemental Table 2.** Uni- and Multivariate Analyses of Prognostic Factors for Overall Survival in Hepatocellular Carcinoma

| **Characteristics** | **Total(N)** | **Univariate analysis** | |  | **Multivariate analysis** | |
| --- | --- | --- | --- | --- | --- | --- |
|  |  | **Hazard ratio (95% CI)** | **P value** |  | **Hazard ratio (95% CI)** | **P value** |
| **ZC3H15** | **303** |  |  |  |  |  |
| Low | 158 | Reference |  |  | Reference |  |
| High | 145 | 1.698 (1.126 - 2.561) | **0.011** |  | 1.747 (1.130 - 2.700) | **0.012** |
| **Gender** | **303** |  |  |  |  |  |
| Female | 91 | Reference |  |  | Reference |  |
| Male | 212 | 0.774 (0.512 - 1.172) | 0.227 |  | 1.015 (0.653 - 1.579) | 0.946 |
| **Age** | **303** |  |  |  |  |  |
| <= 60 | 156 | Reference |  |  | Reference |  |
| > 60 | 147 | 1.320 (0.884 - 1.971) | 0.175 |  | 1.416 (0.919 - 2.181) | 0.114 |
| **BMI** | **303** |  |  |  |  |  |
| <= 25 | 169 | Reference |  |  | Reference |  |
| > 25 | 134 | 0.748 (0.499 - 1.121) | 0.160 |  | 0.691 (0.453 - 1.054) | 0.086 |
| **Pathologic stage** | **303** |  |  |  |  |  |
| Stage I | 153 | Reference |  |  | Reference |  |
| Stage II | 73 | 1.574 (0.915 - 2.708) | 0.102 |  | 1.422 (0.822 - 2.460) | 0.208 |
| Stage III | 75 | 2.951 (1.864 - 4.671) | **< 0.001** |  | 2.351 (1.461 - 3.784) | **< 0.001** |
| Stage IV | 2 | 4.791 (1.147 - 20.015) | **0.032** |  | 3.505 (0.777 - 15.809) | 0.103 |
| **Tumor status** | **303** |  |  |  |  |  |
| Tumor free | 178 | Reference |  |  | Reference |  |
| With tumor | 125 | 2.466 (1.624 - 3.745) | **< 0.001** |  | 2.006 (1.299 - 3.098) | **0.002** |

**Supplemental Table 3.** Uni- and Multivariate Analyses of Prognostic Factors for Disease-Specific Survival in Hepatocellular Carcinoma

| **Characteristics** | **Total (N)** | **Univariate analysis** | |  | **Multivariate analysis** | |
| --- | --- | --- | --- | --- | --- | --- |
|  |  | **Hazard ratio (95% CI)** | **P value** |  | **Hazard ratio (95% CI)** | **P value** |
| **ZC3H15** | **338** |  |  |  |  |  |
| Low | 169 | Reference |  |  | Reference |  |
| High | 169 | 1.591 (0.974 - 2.600) | 0.064 |  | 1.662 (1.000 - 2.762) | 0.050 |
| **Gender** | **338** |  |  |  |  |  |
| Female | 108 | Reference |  |  | Reference |  |
| Male | 230 | 0.814 (0.495 - 1.338) | 0.417 |  | 0.946 (0.557 - 1.608) | 0.838 |
| **Age** | **338** |  |  |  |  |  |
| <= 60 | 168 | Reference |  |  | Reference |  |
| > 60 | 170 | 0.856 (0.527 - 1.391) | 0.531 |  | 0.784 (0.471 - 1.305) | 0.350 |
| **Pathologic stage** | **338** |  |  |  |  |  |
| Stage I | 168 | Reference |  |  | Reference |  |
| Stage II | 83 | 1.571 (0.781 - 3.161) | 0.206 |  | 1.523 (0.756 - 3.069) | 0.239 |
| Stage III | 83 | 4.186 (2.380 - 7.361) | **< 0.001** |  | 4.128 (2.346 - 7.265) | **< 0.001** |
| Stage IV | 4 | 9.176 (2.126 - 39.600) | **0.003** |  | 11.978 (2.691 - 53.313) | **0.001** |

**Supplemental Table 4.** Uni- and Multivariate Analyses of Prognostic Factors for Progression-Free Interval in Hepatocellular Carcinoma

| **Characteristics** | **Total(N)** | **Univariate analysis** | |  | **Multivariate analysis** | |
| --- | --- | --- | --- | --- | --- | --- |
|  |  | **Hazard ratio (95% CI)** | **P value** |  | **Hazard ratio (95% CI)** | **P value** |
| **ZC3H15** | **303** |  |  |  |  |  |
| Low | 158 | Reference |  |  | Reference |  |
| High | 145 | 1.321 (0.957 - 1.823) | 0.090 |  | 1.089 (0.780 - 1.520) | 0.617 |
| **Sex** | **303** |  |  |  |  |  |
| Female | 91 | Reference |  |  | Reference |  |
| Male | 212 | 1.027 (0.722 - 1.461) | 0.881 |  | 1.519 (1.047 - 2.204) | **0.028** |
| **Age** | **303** |  |  |  |  |  |
| <= 60 | 156 | Reference |  |  | Reference |  |
| > 60 | 147 | 0.919 (0.665 - 1.268) | 0.606 |  | 0.741 (0.521 - 1.053) | 0.095 |
| **BMI** | **303** |  |  |  |  |  |
| <= 25 | 169 | Reference |  |  | Reference |  |
| > 25 | 134 | 0.925 (0.670 - 1.277) | 0.636 |  | 0.858 (0.605 - 1.216) | 0.388 |
| **Pathologic stage** | **303** |  |  |  |  |  |
| Stage I | 153 | Reference |  |  | Reference |  |
| Stage II | 73 | 1.809 (1.204 - 2.719) | **0.004** |  | 1.493 (0.984 - 2.266) | 0.060 |
| Stage III | 75 | 2.742 (1.872 - 4.016) | **< 0.001** |  | 1.915 (1.275 - 2.878) | **0.002** |
| Stage IV | 2 | 5.718 (1.382 - 23.650) | **0.016** |  | 3.583 (0.812 - 15.803) | 0.092 |
| **Tumor status** | **303** |  |  |  |  |  |
| Tumor free | 178 | Reference |  |  | Reference |  |
| With tumor | 125 | 12.393 (8.034 - 19.116) | **< 0.001** |  | 13.277 (8.460 - 20.837) | **< 0.001** |

**Supplemental Table 5.** Uni- and Multivariate Analyses of Prognostic Factors for Overall Survival in Lung Adenocarcinoma

| **Characteristics** | **Total(N)** | **Univariate analysis** | |  | **Multivariate analysis** | |
| --- | --- | --- | --- | --- | --- | --- |
|  |  | **Hazard ratio (95% CI)** | **P value** |  | **Hazard ratio (95% CI)** | **P value** |
| **ZC3H15** | **426** |  |  |  |  |  |
| Low | 222 | Reference |  |  | Reference |  |
| High | 204 | 1.375 (0.985 - 1.920) | 0.062 |  | 1.058 (0.751 - 1.492) | 0.746 |
| **Gender** | **426** |  |  |  |  |  |
| Female | 241 | Reference |  |  | Reference |  |
| Male | 185 | 1.023 (0.732 - 1.431) | 0.893 |  | 0.923 (0.658 - 1.296) | 0.643 |
| **Age** | **426** |  |  |  |  |  |
| <= 65 | 219 | Reference |  |  | Reference |  |
| > 65 | 207 | 1.146 (0.821 - 1.599) | 0.422 |  | 1.080 (0.769 - 1.519) | 0.656 |
| **Pathologic stage** | **426** |  |  |  |  |  |
| Stage I | 245 | Reference |  |  | Reference |  |
| Stage II | 100 | 2.074 (1.380 - 3.116) | **< 0.001** |  | 1.942 (1.280 - 2.946) | **0.002** |
| Stage III | 62 | 3.056 (1.978 - 4.723) | **< 0.001** |  | 2.943 (1.892 - 4.575) | **< 0.001** |
| Stage IV | 19 | 4.231 (2.200 - 8.136) | **< 0.001** |  | 3.209 (1.617 - 6.369) | **< 0.001** |
| **Primary therapy outcome** | **426** |  |  |  |  |  |
| PD | 70 | Reference |  |  | Reference |  |
| SD | 37 | 0.247 (0.116 - 0.526) | **< 0.001** |  | 0.235 (0.110 - 0.503) | **< 0.001** |
| CR | 314 | 0.273 (0.189 - 0.394) | **< 0.001** |  | 0.312 (0.212 - 0.457) | **< 0.001** |
| PR | 5 | 0.683 (0.165 - 2.820) | 0.598 |  | 0.674 (0.162 - 2.804) | 0.587 |

**Supplemental Table 6.** Uni- and Multivariate Analyses of Prognostic Factors for Progression-Free Interval in Lung Adenocarcinoma

| **Characteristics** | **Total(N)** | **Univariate analysis** | |  | **Multivariate analysis** | |
| --- | --- | --- | --- | --- | --- | --- |
|  |  | **Hazard ratio (95% CI)** | **P value** |  | **Hazard ratio (95% CI)** | **P value** |
| **ZC3H15** | **426** |  |  |  |  |  |
| Low | 222 | Reference |  |  | Reference |  |
| High | 204 | 1.393 (1.048 - 1.852) | **0.022** |  | 1.065 (0.793 - 1.429) | 0.675 |
| **Gender** | **426** |  |  |  |  |  |
| Female | 241 | Reference |  |  | Reference |  |
| Male | 185 | 1.319 (0.992 - 1.754) | 0.057 |  | 1.287 (0.962 - 1.721) | 0.090 |
| **Age** | **426** |  |  |  |  |  |
| <= 65 | 219 | Reference |  |  | Reference |  |
| > 65 | 207 | 1.106 (0.832 - 1.471) | 0.487 |  | 0.895 (0.662 - 1.209) | 0.469 |
| **Pathologic stage** | **426** |  |  |  |  |  |
| Stage I | 245 | Reference |  |  | Reference |  |
| Stage II | 100 | 1.772 (1.268 - 2.476) | **< 0.001** |  | 1.367 (0.967 - 1.932) | 0.077 |
| Stage III | 62 | 1.939 (1.300 - 2.893) | **0.001** |  | 1.630 (1.082 - 2.454) | **0.019** |
| Stage IV | 19 | 2.294 (1.253 - 4.199) | **0.007** |  | 0.986 (0.517 - 1.879) | 0.966 |
| **Primary therapy outcome** | **426** |  |  |  |  |  |
| PD | 70 | Reference |  |  | Reference |  |
| SD | 37 | 0.198 (0.113 - 0.348) | **< 0.001** |  | 0.185 (0.103 - 0.331) | **< 0.001** |
| CR | 314 | 0.158 (0.115 - 0.216) | **< 0.001** |  | 0.165 (0.117 - 0.232) | **< 0.001** |
| PR | 5 | 0.236 (0.058 - 0.964) | **0.044** |  | 0.268 (0.065 - 1.104) | 0.068 |

**Supplemental Table 7.** Uni- and Multivariate Analyses of Prognostic Factors for Disease-Specific Survival in Lung Adenocarcinoma

| **Characteristics** | **Total(N)** | **Univariate analysis** | |  | **Multivariate analysis** | |
| --- | --- | --- | --- | --- | --- | --- |
|  |  | **Hazard ratio (95% CI)** | **P value** |  | **Hazard ratio (95% CI)** | **P value** |
| **ZC3H15** | **415** |  |  |  |  |  |
| Low | 214 | Reference |  |  | Reference |  |
| High | 201 | 1.490 (1.000 - 2.220) | 0.050 |  | 1.063 (0.704 - 1.604) | 0.771 |
| **Gender** | **415** |  |  |  |  |  |
| Female | 234 | Reference |  |  | Reference |  |
| Male | 181 | 1.047 (0.703 - 1.559) | 0.823 |  | 0.932 (0.624 - 1.392) | 0.730 |
| **Age** | **415** |  |  |  |  |  |
| <= 65 | 213 | Reference |  |  | Reference |  |
| > 65 | 202 | 1.123 (0.755 - 1.669) | 0.567 |  | 1.037 (0.690 - 1.558) | 0.861 |
| **Pathologic stage** | **415** |  |  |  |  |  |
| Stage I | 240 | Reference |  |  | Reference |  |
| Stage II | 96 | 2.622 (1.614 - 4.258) | **< 0.001** |  | 2.232 (1.353 - 3.680) | **0.002** |
| Stage III | 61 | 3.383 (1.998 - 5.730) | **< 0.001** |  | 3.165 (1.849 - 5.417) | **< 0.001** |
| Stage IV | 18 | 5.197 (2.483 - 10.875) | **< 0.001** |  | 3.284 (1.516 - 7.114) | **0.003** |
| **Primary therapy outcome** | **415** |  |  |  |  |  |
| PD | 70 | Reference |  |  | Reference |  |
| SD | 37 | 0.231 (0.104 - 0.515) | **< 0.001** |  | 0.217 (0.096 - 0.487) | **< 0.001** |
| CR | 303 | 0.177 (0.117 - 0.269) | **< 0.001** |  | 0.207 (0.134 - 0.320) | **< 0.001** |
| PR | 5 | 0.348 (0.048 - 2.533) | 0.298 |  | 0.359 (0.049 - 2.634) | 0.314 |

**Supplemental Table 8.** Uni- and Multivariate Analyses of Prognostic Factors for Overall Survival in BRAC

| **Characteristics** | **Total(N)** | **Univariate analysis** | |  | **Multivariate analysis** | |
| --- | --- | --- | --- | --- | --- | --- |
|  |  | **Hazard ratio (95% CI)** | **P value** |  | **Hazard ratio (95% CI)** | **P value** |
| **ZC3H15** | **1,080** |  |  |  |  |  |
| Low | 808 | Reference |  |  | Reference |  |
| High | 272 | 1.444 (1.017 - 2.051) | **0.040** |  | 1.679 (1.168 - 2.413) | **0.005** |
| **Age** | **1,080** |  |  |  |  |  |
| <=60 | 600 | Reference |  |  | Reference |  |
| >60 | 480 | 2.035 (1.472 - 2.814) | **< 0.001** |  | 2.019 (1.441 - 2.829) | **< 0.001** |
| **Tumor stage** | **1,075** |  |  |  |  |  |
| Stage X | 13 | Reference |  |  | Reference |  |
| Stage II | 619 | 0.412 (0.188 - 0.904) | **0.027** |  | 0.387 (0.173 - 0.864) | **0.021** |
| Stage I | 181 | 0.242 (0.099 - 0.593) | **0.002** |  | 0.219 (0.088 - 0.544) | **0.001** |
| Stage III | 244 | 0.708 (0.315 - 1.592) | 0.404 |  | 0.703 (0.306 - 1.614) | 0.406 |
| Stage IV | 18 | 2.813 (1.111 - 7.123) | **0.029** |  | 2.299 (0.898 - 5.887) | 0.083 |

**Supplemental Table 9.** Uni- and Multivariate Analyses of Prognostic Factors for Progression-Free Interval in LUSC

| **Characteristics** | **Total(N)** | **Univariate analysis** | |  | **Multivariate analysis** | |
| --- | --- | --- | --- | --- | --- | --- |
|  |  | **Hazard ratio (95% CI)** | **P value** |  | **Hazard ratio (95% CI)** | **P value** |
| **ZC3H15** | **498** |  |  |  |  |  |
| Low | 157 | Reference |  |  | Reference |  |
| High | 341 | 1.523 (1.037 - 2.237) | **0.032** |  | 1.502 (1.018 - 2.214) | **0.040** |
| **Age** | **489** |  |  |  |  |  |
| <=60 | 107 | Reference |  |  | Reference |  |
| >60 | 382 | 0.986 (0.666 - 1.462) | 0.946 |  | 1.132 (0.756 - 1.694) | 0.548 |
| **Gender** | **498** |  |  |  |  |  |
| Female | 131 | Reference |  |  | Reference |  |
| Male | 367 | 1.153 (0.788 - 1.687) | 0.464 |  | 1.084 (0.736 - 1.596) | 0.685 |
| **Tumor_stage** | **498** |  |  |  |  |  |
| Stage I | 245 | Reference |  |  | Reference |  |
| Stage II | 162 | 1.514 (1.038 - 2.207) | **0.031** |  | 1.442 (0.987 - 2.107) | 0.059 |
| Stage III | 84 | 2.199 (1.444 - 3.347) | **< 0.001** |  | 2.214 (1.454 - 3.371) | **< 0.001** |
| Stage IV | 7 | 2.098 (0.511 - 8.617) | 0.304 |  | 1.850 (0.449 - 7.625) | 0.395 |

**Supplemental Table 10**. Uni- and Multivariate Analyses of Prognostic Factors for Progression-Free Interval in HNSC

| **Characteristics** | **Total(N)** | **Univariate analysis** | |  | **Multivariate analysis** | |
| --- | --- | --- | --- | --- | --- | --- |
|  |  | **Hazard ratio (95% CI)** | **P value** |  | **Hazard ratio (95% CI)** | **P value** |
| **ZC3H15** | **479** |  |  |  |  |  |
| Low | 244 | Reference |  |  | Reference |  |
| High | 235 | 1.449 (1.082 - 1.941) | **0.013** |  | 1.450 (1.079 - 1.950) | **0.014** |
| **Gender** | **479** |  |  |  |  |  |
| Female | 129 | Reference |  |  | Reference |  |
| Male | 350 | 1.040 (0.746 - 1.449) | 0.817 |  | 0.926 (0.654 - 1.311) | 0.665 |
| **Age** | **479** |  |  |  |  |  |
| <= 60 | 236 | Reference |  |  | Reference |  |
| > 60 | 243 | 1.130 (0.846 - 1.511) | 0.408 |  | 1.208 (0.898 - 1.625) | 0.211 |
| **Alcohol history** | **479** |  |  |  |  |  |
| No | 157 | Reference |  |  | Reference |  |
| Yes | 322 | 1.371 (0.991 - 1.895) | 0.056 |  | 1.423 (1.015 - 1.996) | **0.041** |
| **Clinical stage** | **479** |  |  |  |  |  |
| Stage I | 19 | Reference |  |  | Reference |  |
| Stage II | 92 | 1.086 (0.455 - 2.593) | 0.853 |  | 1.134 (0.473 - 2.718) | 0.778 |
| Stage III | 100 | 1.141 (0.480 - 2.715) | 0.766 |  | 1.157 (0.482 - 2.775) | 0.744 |
| Stage IV | 268 | 1.371 (0.603 - 3.121) | 0.452 |  | 1.339 (0.585 - 3.065) | 0.489 |

**Supplemental Table 11.** Uni- and Multivariate Analyses of Prognostic Factors for Disease-Specific Survival in HNSC

| **Characteristics** | **Total(N)** | **Univariate analysis** | |  | **Multivariate analysis** | |
| --- | --- | --- | --- | --- | --- | --- |
|  |  | **Hazard ratio (95% CI)** | **P value** |  | **Hazard ratio (95% CI)** | **P value** |
| **ZC3H15** | **455** |  |  |  |  |  |
| Low | 230 | Reference |  |  | Reference |  |
| High | 225 | 1.459 (1.025 - 2.079) | **0.036** |  | 1.462 (1.020 - 2.096) | **0.039** |
| **Gender** | **455** |  |  |  |  |  |
| Female | 119 | Reference |  |  | Reference |  |
| Male | 336 | 0.936 (0.629 - 1.393) | 0.746 |  | 0.851 (0.561 - 1.292) | 0.449 |
| **Age** | **455** |  |  |  |  |  |
| <= 60 | 227 | Reference |  |  | Reference |  |
| > 60 | 228 | 1.126 (0.793 - 1.599) | 0.506 |  | 1.165 (0.814 - 1.666) | 0.404 |
| **Alcohol history** | **455** |  |  |  |  |  |
| No | 145 | Reference |  |  | Reference |  |
| Yes | 310 | 1.224 (0.828 - 1.808) | 0.310 |  | 1.263 (0.841 - 1.897) | 0.260 |
| **Clinical stage** | **455** |  |  |  |  |  |
| Stage I | 19 | Reference |  |  | Reference |  |
| Stage II | 85 | 0.935 (0.352 - 2.483) | 0.893 |  | 0.981 (0.368 - 2.613) | 0.969 |
| Stage III | 93 | 1.051 (0.402 - 2.748) | 0.919 |  | 1.065 (0.403 - 2.811) | 0.899 |
| Stage IV | 258 | 1.164 (0.470 - 2.880) | 0.743 |  | 1.145 (0.460 - 2.848) | 0.771 |
