# Supplementary material for: ZC3H15 in human tumors: a pan-cancer exploration of its clinical significance, molecular function, and association with the tumor immune microenvironment
Source: Front Immunol. 2026 Oct 5;17:1901516. doi: 10.3389/fimmu.2026.1901516 (PMC13677929; doi:10.3389/fimmu.2026.1901516)
Supplement: Supplementary file 4 [file DataSheet3.zip › raw data MS (ID 1901516)-3/Supplementary Figure 7A,B,C/Figure S7A Flow cytometry (MHCC97H)/Gating.pdf]

In this study, propidium iodide (PI) was used to stain DNA, following a standard protocol for flow cytometric cell cycle analysis. Below, we present the corresponding gating strategies.

1. Core Gating Strategy in Flow Cytometric Cell Cycle Analysis

(1) The first step is to analyze an FSC-A versus SSC-A scatter plot to gate the entire target cell population and exclude cell debris and artifacts. (2) The second step consists of gating single cells using an FSC-A vs. FSC-H scatter plot to exclude cell doublets or aggregates, thereby avoiding erroneous DNA content quantification due to such events. (3) The third step is to partition the cell population based on the PI fluorescence histogram acquired in linear mode into three subpopulations: G0/G1, S, and G2/M.

2. Explanation of Fluorescent Pigments

PI is an intercalating fluorescent nucleic acid dye that is excited by a 488-nm laser and emits red fluorescence, which is typically detected in the FL2 or PE channels.

3. The gating strategies were used in the study.

3.1 siNC

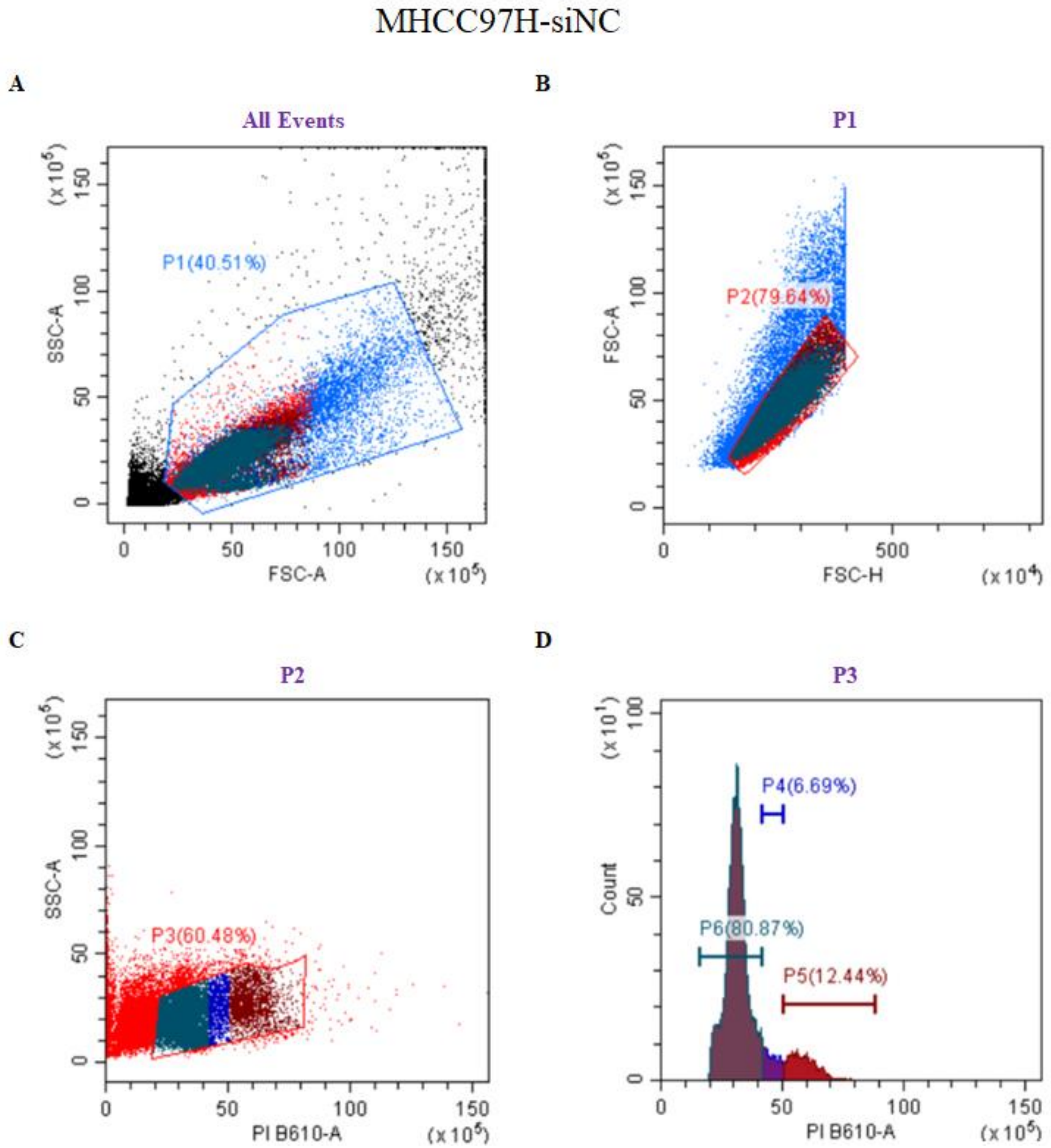

Note: (A) The first step is to analyze an FSC-A versus SSC-A scatter plot to gate the entire target cell population and exclude cell debris and artifacts. (B) The second step consists of gating single cells using an FSC-A vs. FSC-H scatter plot to exclude cell doublets or aggregates, thereby avoiding erroneous DNA content quantification due to such events. (C, D) The third step is to partition the cell population based on the PI fluorescence histogram acquired in linear mode into three subpopulations: G0/G1, S, and G2/M.

3.2 siZC3H15#1

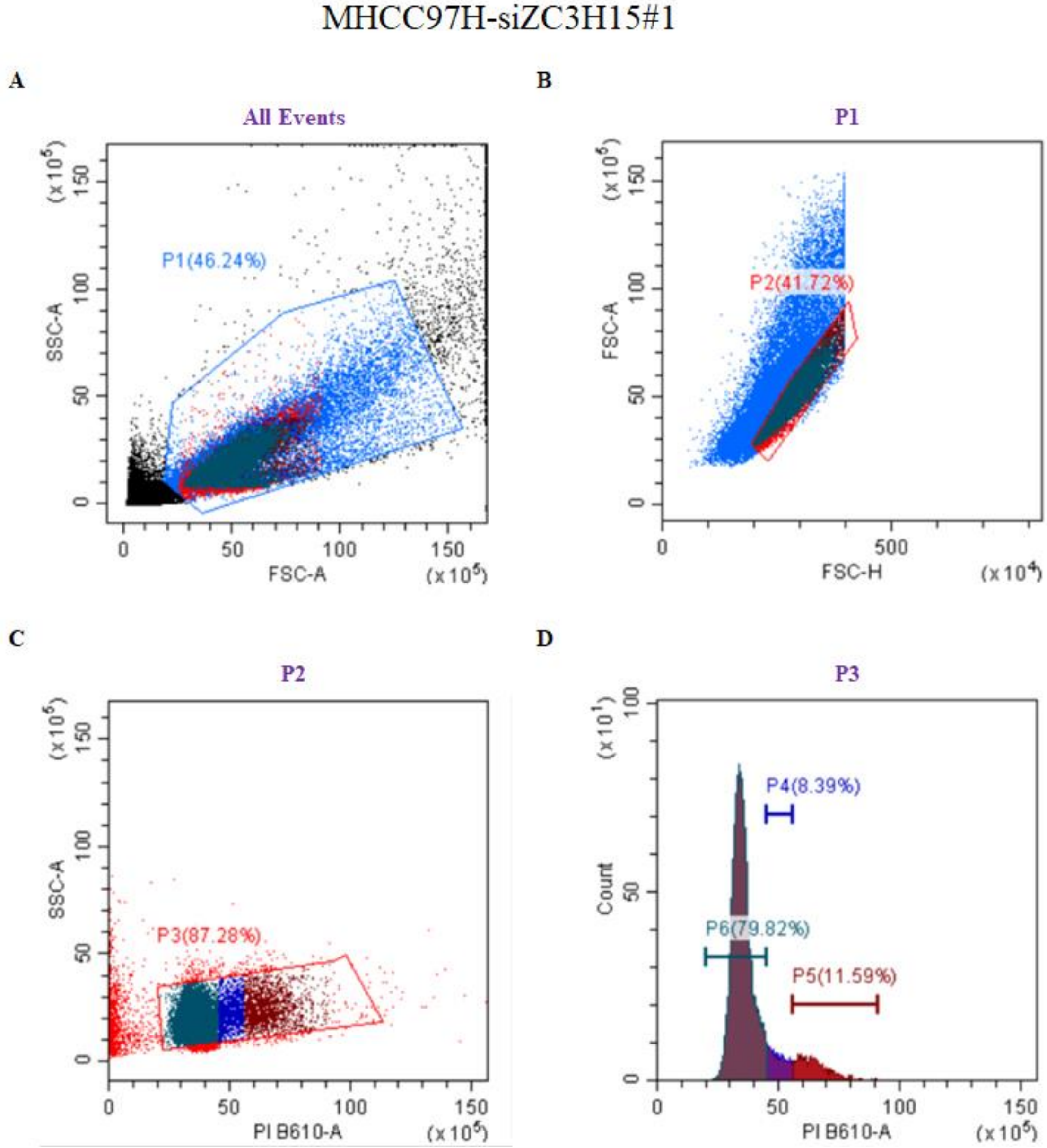

Note: (A) The first step is to analyze an FSC-A versus SSC-A scatter plot to gate the entire target cell population and exclude cell debris and artifacts. (B) The second step consists of gating single cells using an FSC-A vs. FSC-H scatter plot to exclude cell doublets or aggregates, thereby avoiding erroneous DNA content quantification due to such events. (C, D) The third step is to partition the cell population based on the PI fluorescence histogram acquired in linear mode into three subpopulations: G0/G1, S, and G2/M.

3.3 siZC3H15#2

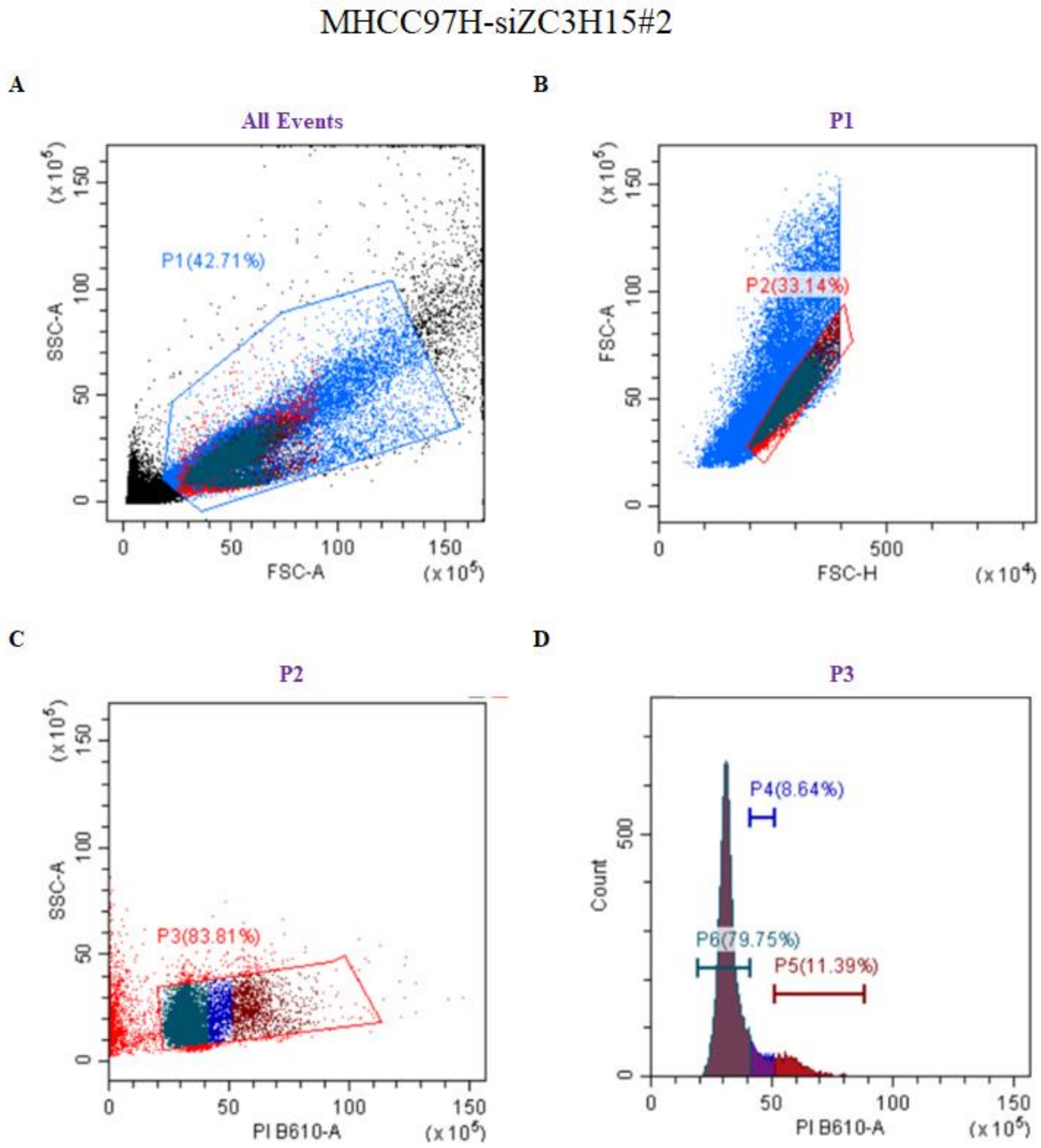

Note: (A) The first step is to analyze an FSC-A versus SSC-A scatter plot to gate the entire target cell population and exclude cell debris and artifacts. (B) The second step consists of gating single cells using an FSC-A vs. FSC-H scatter plot to exclude cell doublets or aggregates, thereby avoiding erroneous DNA content quantification due to such events. (C, D) The third step is to partition the cell population based on the PI fluorescence histogram acquired in linear mode into three subpopulations: G0/G1, S, and G2/M.

3.4 siZC3H15#3

MHCC97H-siZC3H15#3

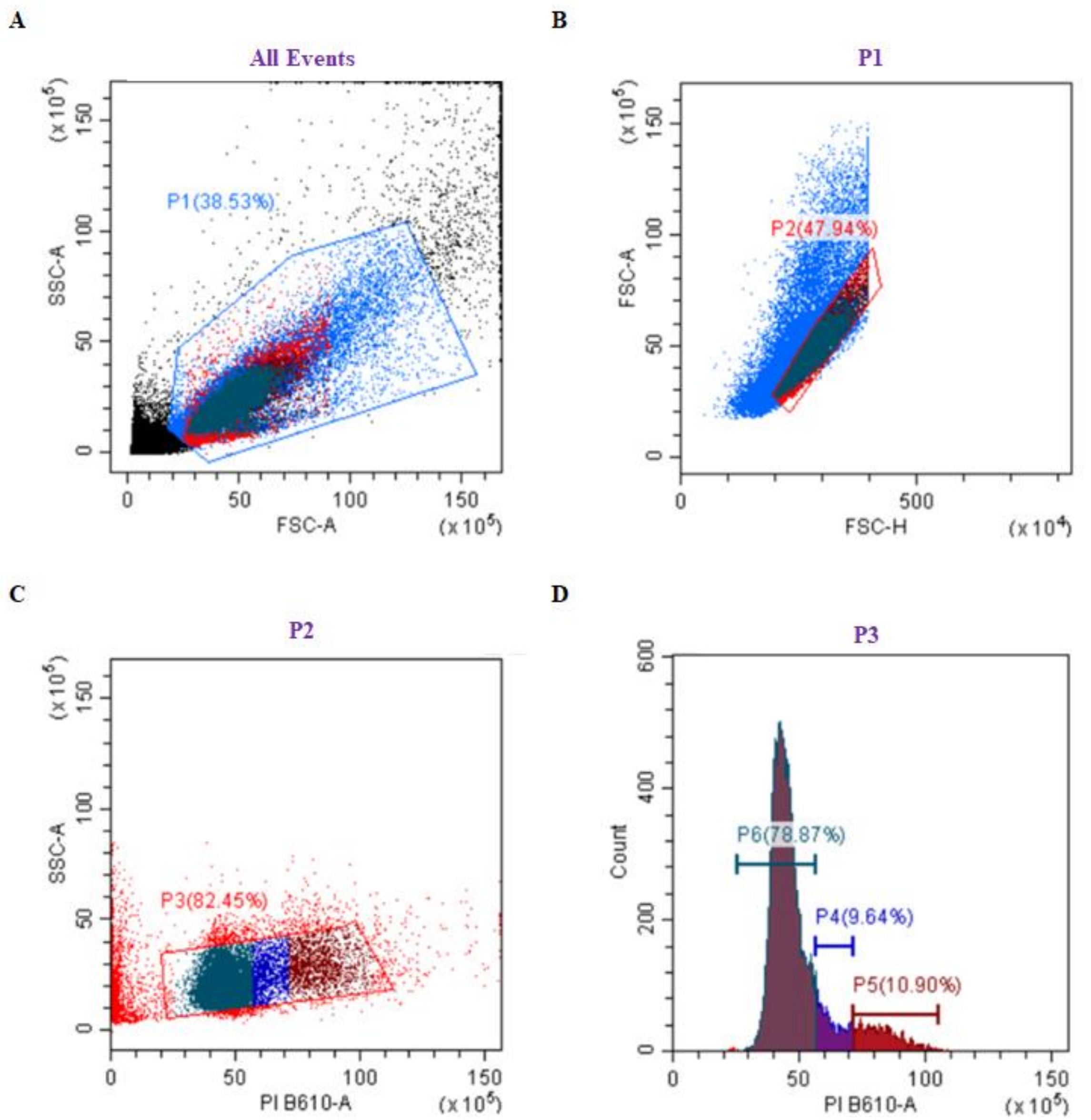

Note: (A) The first step is to analyze an FSC-A versus SSC-A scatter plot to gate the entire target cell population and exclude cell debris and artifacts. (B) The second step consists of gating single cells using an FSC-A vs. FSC-H scatter plot to exclude cell doublets or aggregates, thereby avoiding erroneous DNA content quantification due to such events. (C, D) The third step is to partition the cell population based on the PI fluorescence histogram acquired in linear mode into three subpopulations: G0/G1, S, and G2/M.
